# Foxd4l1.1 negatively regulates transcription of neural repressor *ventx1.1* during neuroectoderm formation in *Xenopus* embryos

**DOI:** 10.1038/s41598-020-73662-4

**Published:** 2020-10-08

**Authors:** Shiv Kumar, Zobia Umair, Vijay Kumar, Santosh Kumar, Unjoo Lee, Jaebong Kim

**Affiliations:** 1 Department of Biochemistry, Institute of Cell Differentiation and Aging, College of Medicine, Hallym University, Gangwon-Do, Chuncheon, 24252 Republic of Korea; 2 Department of Electrical Engineering, Hallym University, Gangwon-Do, Chuncheon, 24252 Republic of Korea

**Keywords:** Biochemistry, Developmental biology, Molecular biology

## Abstract

Neuroectoderm formation is the first step in development of a proper nervous system for vertebrates. The developmental decision to form a non-neural ectoderm versus a neural one involves the regulation of BMP signaling, first reported many decades ago. However, the precise regulatory mechanism by which this is accomplished has not been fully elucidated, particularly for transcriptional regulation of certain key transcription factors. BMP4 inhibition is a required step in eliciting neuroectoderm from ectoderm and Foxd4l1.1 is one of the earliest neural genes highly expressed in the neuroectoderm and conserved across vertebrates, including humans. In this work, we focused on how Foxd4l1.1 downregulates the neural repressive pathway. Foxd4l1.1 inhibited BMP4/Smad1 signaling and triggered neuroectoderm formation in animal cap explants of *Xenopus* embryos. Foxd4l1.1 directly bound within the promoter of endogenous neural repressor *ventx1.1* and inhibited *ventx1.1* transcription. Foxd4l1.1 also physically interacted with Xbra in the nucleus and inhibited Xbra-induced *ventx1.1* transcription. In addition, Foxd4l1.1 also reduced nuclear localization of Smad1 to inhibit Smad1-mediated *ventx1.1* transcription. Foxd4l1.1 reduced the direct binding of Xbra and Smad1 on *ventx1.1* promoter regions to block Xbra/Smad1-induced synergistic activation of *ventx1.1* transcription. Collectively, Foxd4l1.1 negatively regulates transcription of a neural repressor *ventx1.1* by multiple mechanisms in its exclusively occupied territory of neuroectoderm, and thus leading to primary neurogenesis. In conjunction with the results of our previous findings that *ventx1.1* directly represses *foxd4l1.1*, the reciprocal repression of *ventx1.1* and *foxd4l1.1* is significant in at least in part specifying the mechanism for the non-neural versus neural ectoderm fate determination in *Xenopus* embryos.

## Introduction

Central to vertebrate embryogenesis, during the gastrula stage of embryogenesis, neuroectoderm develops from the naive ectoderm, giving rise to the neural plate, neural tube, and the neural crest. It is known that the vertebrate neuroectoderm develops in response to the dorsal mesoderm (the Spemann organizer)^1–4^ with BMP signaling becoming inhibited^5–7^. In this regard, inhibition of BMP4 signaling by various BMP4-antagonists such as the dominant-negative BMP receptor (DNBR), noggin and chordin cause dorso-anteriorization and neuroectoderm formation of embryos^5–8^. These observations have raised the possibility that the neuroectoderm formation is a default pathway instead of a previously proposed inductive event by instructive signals emanating from the organizer^9,10^ and for non-neural ectoderm formation, there are instructive signals that include BMP4^11^ with the BMP4/Smad1 axis leading to a negative regulation of the neuroectoderm^5–11^. In support of this, BMP target transcription factors including Gata1b, Msx1 and Ventx1.1 have been shown to be repressive transcription factors (TFs) of neural ectoderm^12–15^. Despite these findings, although the inhibition of BMP signals for eliciting the neuroectoderm has been well documented at the extracellular level, the pathway and its target genes in the neuroectoderm remain undefined, particularly at the transcription level.

BMP4/Smad1 is known to interact with FGF/Xbra to induce the expression of an endogenous neural repressor transcription factor (TF) Ventx1.1^15,16^. Ventx1.1 is a homeobox transcription repressor which inhibits the expression of organizer-specific and neural genes, including *gsc*, *chordin*, *noggin, ncam*, *ngnr*, *zic3* and *foxd4l1.1*^17,18^. Dorsal expression of *ventx1.1* results in the headless phenotype and neural inhibition in *Xenopus* embryos^17,18^. FGF signaling is involved in germ layer specification and anterior–posterior patterning of neural tissues in vertebrates. Specifically, FGF signaling leads to mesodermal formation through activation of an autocatalytic loop of FGF/Ras/Xbra/AP1 in *Xenopus*^19,20^. Studies have also revealed the opposing roles of FGF signaling that regulate neuroectoderm formation during gastrula^21–24^. FGF/MAPK activation was shown to inhibit BMP4/Smad1 signaling and promote neuroectoderm formation by phosphorylating the linker region of Smad1, resulting in the cytoplasmic retention of Smad1^21–24^. On the other hand, signaling by both BMP4/Smad1 and FGF/Xbra had a synergistic role in activating *ventx1.1* transcription to inhibit neuroectoderm formation^15,16^. The details of inhibitory gene regulatory network and transcriptional mechanism of the neural repressor gene *ventx1.1* in neuroectoderm region await elucidation.

Once the neural ectoderm is formed and neural transcription factors (nTFs) are expressed, some of the earliest expressed nTFs need to prevent cells from reverting to a non-neural fate^25–27^. The primary neuroectoderm expresses various transcription factors that may inhibit it from becoming the epidermis^25,28^. Foxd4l1.1 of the forkhead box (fox) family of transcription factors (also known as *Xenopus* fork-head expressed in the dorsal lip, *foxd4l1.1*, with other designations being *xflip, foxd4-like1*, *foxd4l1*, *xfd-12* and *foxd5b*) is reported as one of the earliest neural genes and is evolutionally conserved and expressed in neuroectoderm across vertebrates including humans^29–31^. *Foxd4l1.1* is known to actively participate in various developmental events, such as immature neuroectoderm fate maintenance, neural plate formation and neural differentiation. It has been shown that the ectopic expression of DNBR stimulates *foxd4l1.1* mRNA expression level in animal cap explants^18,32–34^. Further, transition of ectoderm-neuroectoderm is regulated by fine-tuning of several transcription factors such as *foxd4l1.1*, *zic2/3*, *sox1-3/d* and *xiro1-3*^25,35^. Foxd4l1.1 has been documented to increase the expression of neural specific genes, including *sox2/3*, *geminin*, *n-tubulin*, *ncam*, and *neuroD* in a dose-dependent manner, leading to neural differentiation^36^. Also, ectopic expression of *foxd4l1.1* inhibits BMP4/Smad1 signaling and leads to neural differentiation and neuroectoderm formation^34^.

We have previously shown that *foxd4l1.1* inhibits the promoter activity of *ventx1.1* and promotes primary neurogenesis in *Xenopus* embryos^15^. A study documented that *foxd4l1.1* and its engrailed (repressor) conjugated construct (*EnRfoxd4l1.1*) induce the expression of neural markers such as *xngnr1*, *n-tubulin*, *geminin* and *xnr3* in animal cap explants^37^. However, the molecular details for *foxd4l1.1* in ectoderm-neuroectodem specification and *foxd4l1.1*-mediated BMP4/Smad1 inhibition, leading to neuroectoderm formation, remain to be elaborated.

In the present study, we focused on the mechanisms of reciprocally exclusive germ-layer (ectoderm, mesoderm and neuroectoderm) specification in early vertebrate embryogenesis. In the mesoderm region, both BMP/Smad1 and FGF/Xbra synergistically upregulate transcription of neural repressor *ventx1.1* and inhibit neuroectoderm formation in *Xenopus* embryos^15,18^. On the other hand, the neuroectoderm region may require a neuroectoderm specific repressor in order to inhibit *ventx1.1* expression. In this work, we found that *foxd4l1.1* and *engrailed-foxd4l1.1* (*EnRfoxd4l1.1*) negatively regulated BMP4/Smad1 signaling and inhibited *ventx1.1* expression. It is also shown that Foxd4l1.1 directly binds to the proximal region of endogenous *ventx1.1* promoter and inhibits *ventx1.1* transcription during neuroectoderm formation. Foxd4l1.1 also inhibits the direct binding of Xbra-Smad1 to block the synergistic activation of *ventx1.1* transcription. This study suggests that neuroectoderm specific repressor Foxd4l1.1 inhibits expression of the neural repressive transcription factor *ventx1.1* to commit and maintain the neuroectoderm fate, obviating mesoderm commitment during germ-layer specification of *Xenopus* embryos.

## Results

### Foxd4l1.1 inhibits BMP4 and its target gene expression

**Figure 1:**
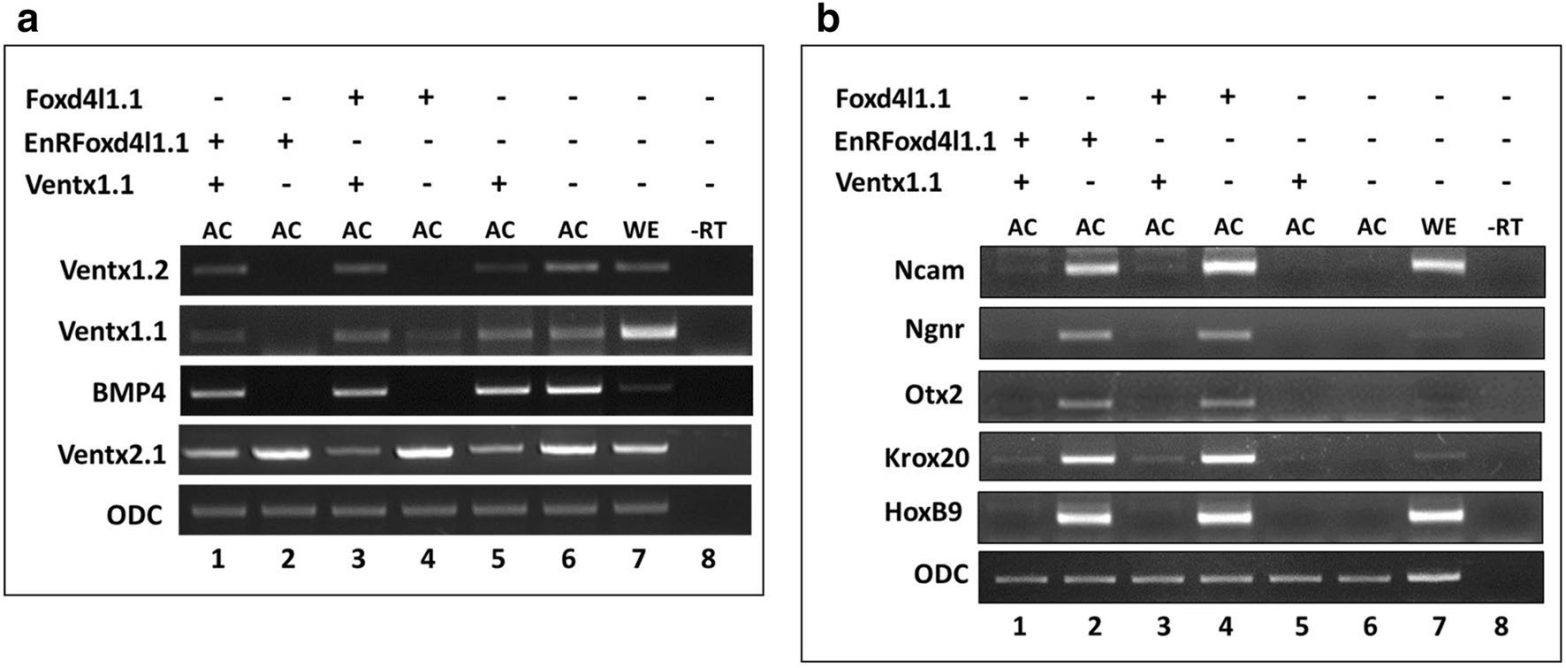
Ectopic expression of *foxd4l1.1* negatively regulates *ventx1.1* transcription in animal cap explants of *Xenopus*. *EnRfoxd4l1.1* (280 pg/embryos) and HA-*foxd4l1.1* (3 ng/embryos) were injected at the one-cell stage and the animal cap were dissected at stage 8 to grow until stage 11 (**a**) and 24 (**b**). The expression profiles of marker genes were analyzed by RT-PCR. No RT (no reverse transcriptase added) served as a negative control while WE (whole embryos) were a positive control.

*Foxd4l1.1* is an early neural transcription factor that contains three distinct domains, each displaying different activities. The three domains are (i) the N-terminal activation domain (the “acidic blob”), (ii) the winged-helix domain (the DNA binding domain) and (iii) the C-terminal repressor domain (Region-II and P/A/Q)^37^. We examined wild type *foxd4l1.1* and *Drosophila* repressor domain (engrailed repressor domain) fused-*foxd4l1.1* (*EnRfoxd4l1.1*) on whether they inhibit *ventx1.1* expression in animal cap explants. *EnRfoxd4l1.1* is the same as *EnRfoxd5a* reported by Sullivan et al.^37^ RT-PCR results indicated that both *foxd4l1.1* and *EnRfoxd4l1.1* suppressed expression of ventral genes, including *bmp4*, *ventx1.1 (PV.1),* and *ventx1.2 (Xvent1)* at stage 11 (Fig. 1a), while *foxd4l1.1* and *EnRfoxd4l1.1* increased expression of neural genes, namely *otx2, krox20*, *hoxB9*, *ncam* and *ngnr* at stage 24 (Fig. 1b). Unexpectedly, expression of *ventx2.1 (Xvent2)* was increased by *foxd4l1.1* and *EnRfoxd4l1.1* (Fig. 1a, lane 2 and 4 vs. lane6, Fig. s1 (RT-qPCR)). Conversely, overexpressed *ventx1.1* reduced expression of ventral genes, *ventx1.1, ventx1.2* and *ventx2.1*, less affecting *bmp4* expression in animal cap explants (Fig. 1a, lane 5 vs. lane 6). *Ventx1.1* also suppressed *foxd4l1.1* and *EnRfoxd4l1.1*-induced expression of neural genes, including *ncam*, *ngnr*, *otx20*, *krox20* and *hoxB9* in animal cap explants (Fig. 1b, lane1 vs. lane2 and lane3 vs. lane4). These results collectively suggested that *foxd4l1.1* inhibits BMP4 signaling and expression of its downstream target genes, leading to neuroectoderm formation. Additionally, *foxd4l1.1* and *ventx1.1* oppositely regulated *ventx2.1* expression in animal explants (Fig. 1a, lane 2 and 4 vs. lane 1, 3 and 5, Fig. s1 (RT-qPCR)). This was an unexpected result as *ventx2.1* is supposed to be induced by BMP4. At the present time, we are not able to explain the reason why *foxd4l1.1* and *EnRfoxd4l1.1* upregulated *ventx2.1* even though it inhibits BMP4 expression. We can assume that the opposite regulation of *ventx2.1* by *foxd4l1.1* and *ventx1.1* may be related to the opposite role of Foxd4l1.1 and Ventx1.1 in neuroectoderm and ectoderm specification as well as in later differentiation of those cells such as neural crest formation. However, details of a fine-tuning regulation and role of ventx2.1 in this context remains unknown.

### Promoter region of *ventx1.1* contains a negative cis-acting element for binding to Foxd4l1.1

**Figure 2:**
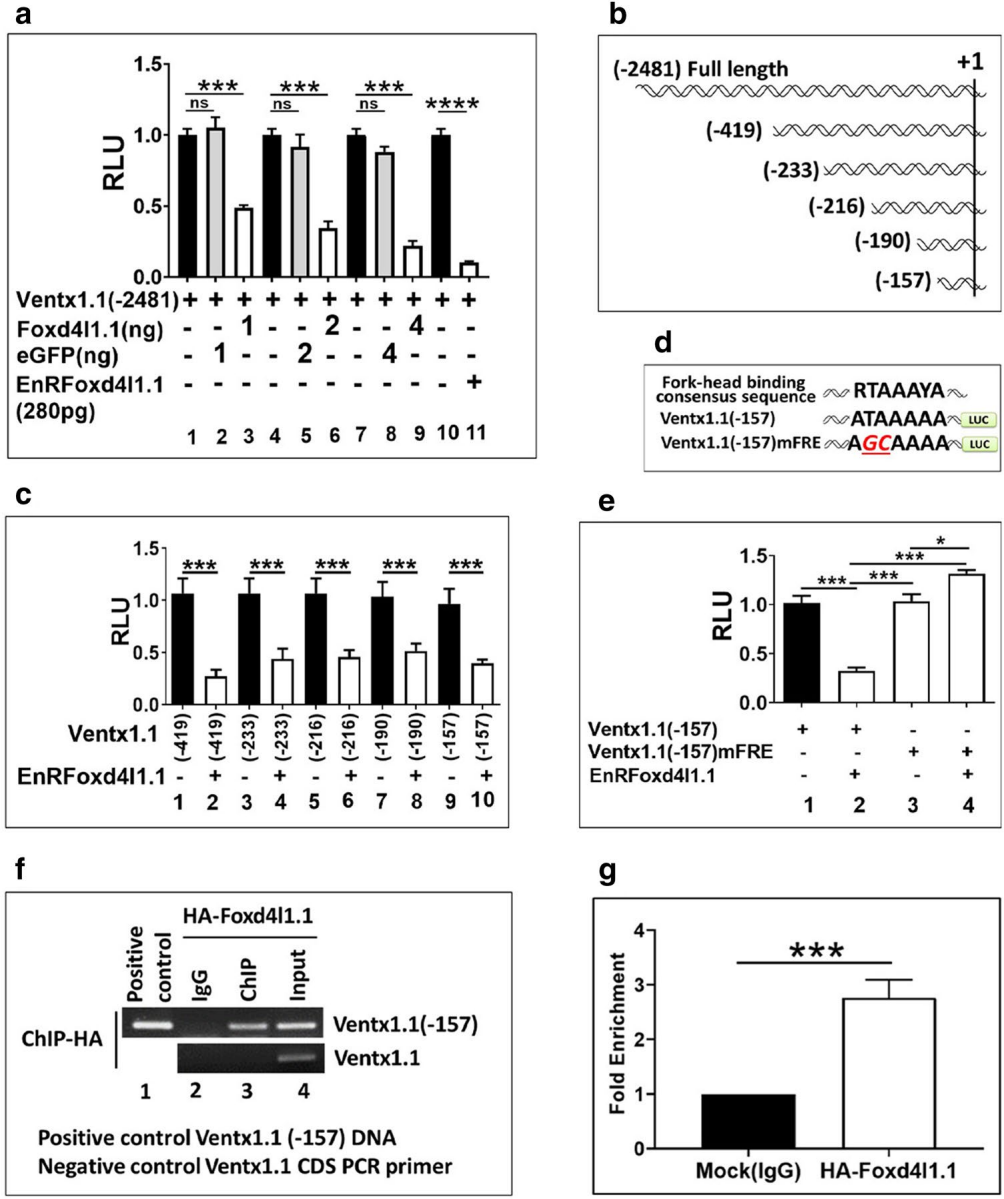
Identification of Foxd4l1.1 response elements within the 5′-flanking region of the *ventx1.1* promoter. All DNA and mRNAs were injected at the one-cell stage, animal-caps dissected at stage 8 and experiments were performed at stage 11 of *Xenopus* embryos. (**a**) *Ventx1.1* (− 2481) promoter (40 pg/embryos) injected with and without *foxd4l1.1* (1, 2 and 4 ng/ embryo), *eGFP* (1, 2 and 4 ng/ embryo) as control in a dose-dependent manner and *EnRfoxd4l1.1* (280 pg/ embryo) to perform the reporter gene assay. (**b**, **c**) Different serially-deleted *ventx1.1* promoter (40 pg/embryo) co-injected with and without *EnRfoxd4l1.1* (280 pg/embryos) to measure the relative promoter activity. (**d**) *ventx1.1* (− 157)mFRE promoter constructs are depicted. (**e**) *Ventx1.1* (− 157)mFRE and *ventx1.1* (− 157) promoter constructs were co-injected with and without *EnRfoxd4l1.1.* (**f**–**g**) HA-*foxd4l1.1* (3 ng/embryo) mRNA injected to perform ChIP-PCR assay with anti-HA antibody (Fold Enrichment Method used to normalize ChIP-qPCR). *Ventx1.1* coding region primers used for RT-PCR as a negative control. All relative promoter activity data are shown as the mean ± SE.

Our previous study demonstrated that the ectopic expression of *foxd4l1.1* inhibits promoter activity of *ventx1.1*^15^, suggesting that the promoter of *ventx1.1* may contain cis-acting response elements for Foxd4l1.1. To delineate this and to define the forkhead response element(s) (FRE) within the *ventx1.1* promoter, *ventx1.1* (− 2481) promoter construct was injected with and without *foxd4l1.1* (wild-type) construct at the one-cell stage at various dosages (1–4 ng) and the reporter gene activity assay was performed. Increasing levels of *foxd4l1.1* reduced the relative promoter activity of *ventx1.1* (2481) at up to 2 to 3.5-fold in a dose-dependent manner (Fig. 2a, bar1 vs 3, bar4 vs 6 and bar 7 vs 9, eGFP control embryos (1–4 ng) in Fig. s2). To enhance the repressor activity of Foxd4l1.1, the repressor form of *foxd4l1.1* (*EnRfoxd4l1.1*, 280 pg) was then examined. *Ventx1.1* (− 2481) promoter construct was co-injected with and without the aforementioned repressor form of *foxd4l1.1* (*EnRfoxd4l1.1*, 280 pg) at the one-cell stage and the reporter gene activity was measured at stage 11. *EnRfoxd4l1.1* remarkably decreased the relative promoter activity as well as the relative mRNA levels of *ventx1.1* at up to eightfold compared with those without *EnRfoxd4l1.1* (Fig. 2a, bar 10 vs 11). Results showed that *EnRfoxd4l1.1* (280 pg/embryos) more efficiently inhibited the relative promoter activity of *ventx1.1* compared to those of wild type *foxd4l1.1* (1–4 ng/embryos, Fig. 2a). Additionally, the results strongly indicated that *ventx1.1* promoter region may contain direct cis-acting FRE(s) for Foxd4l1.1 to inhibit *ventx1.1* transcription. To identify the location of FRE(s) within the *ventx1.1* promoter region, we generated and examined serially-deleted promoter constructs of *ventx1.1* with and without *EnRfoxd4l1.1* (Fig. 2b,c). It is shown that *EnRfoxd4l1.1* decreased the relative promoter activity of all serially-deleted promoter constructs of *ventx1.1* at stage 11 (Fig. 2c). These indicated that the smallest construct of *ventx1.1* (− 157) promoter still contained the putative consensus FRE. A previous study indicated putative human fork-head proteins binding consensus sequences (RTAAAYA; FRE) within the promoter region of Fox family-targeted genes^38^, and *ventx1.1* (− 157) promoter region also contains one of these putative FREs, ATAAAA, referred to as FRE1 (− 82 to − 76 region of *ventx1.1* promoter). To determine whether the putative consensus FRE1 actually functions as a Foxd4l1.1 response element within the *ventx1.1* promoter region, we mutated the sequence (ATAAAAA to AGCAAAA) within the *ventx1.1* (− 157) promoter construct as shown in Fig. 2d. The effect of FRE1-mutated *ventx1.1* (− 157) mFRE1 and the unaltered *ventx1.1* (− 157) constructs were then examined in the same assay. As shown, *foxd4l1.1*-mediated reduction of *ventx1.1* (− 157) was reversed in the FRE1-mutated ventx1.1 (− 157) mFRE1 (Fig. 2e, bar2 vs bar4), indicating that *ventx1.1* (− 157) promoter contains the cis-acting FRE1 for Foxd4l1.1, which is required in inhibition of *ventx1.1* transcription. We next asked whether Foxd4l1.1 directly bound within the proximal region of *ventx1.1* promoter. HA*-foxd4l1.1* mRNA was injected at the one-cell stage and a ChIP-PCR assay was performed at stage 11 in *Xenopus* whole embryos. The results showed that Foxd4l1.1 directly binds within the proximal region of endogenous *ventx1.1* promoter (Fig. 2f (ChIP-PCR), 2 g(ChIP-qPCR)). We concluded that the *ventx1.1* promoter region contains a cis-acting FRE1 (ATAAAA, − 82 to − 76 region of the promoter; highlighted as black in Fig. s3) where Foxd4l1.1 directly binds and inhibits *ventx1.1* transcription.

### C-terminal of Foxd4l1.1 physically interacts with Xbra to inhibit *xbra*-induced transcription of *ventx1.1*

**Figure 3:**
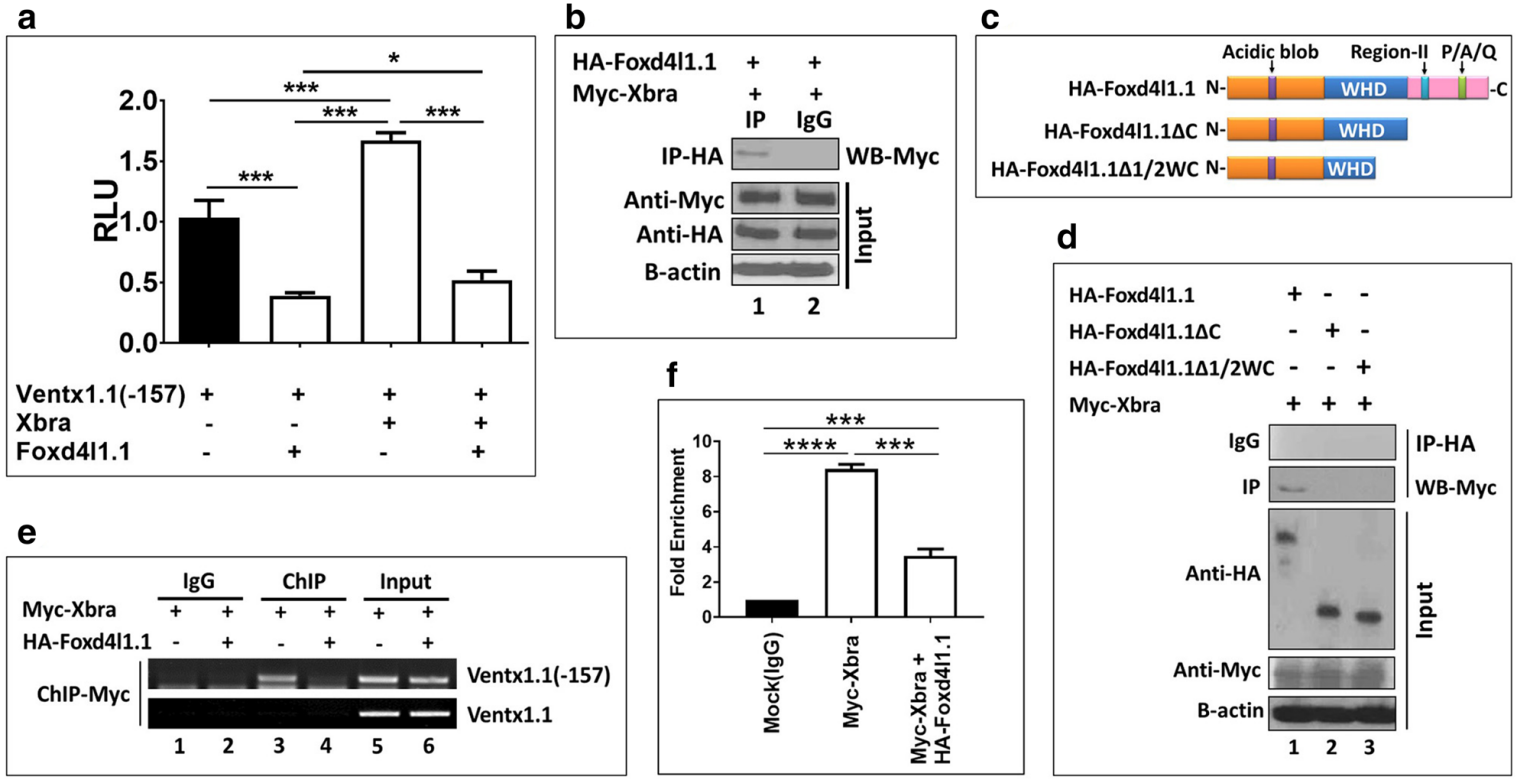
C-terminal of *foxd4l1.1* physically interacts with Xbra and inhibits *xbra*-induced transcription of *ventx1.1*. (**a**) *ventx1.1* (− 157) promoter were co-injected with and without *xbra* and *foxd4l1.1* in different groups to measure the relative promoter activity. (**b**, **d**) Co-immunoprecipitation assay was performed to describe the interaction of Xbra with HA-Foxd4l1.1 and different truncations of HA-Foxd4l1.1. Immunoprecipitation was performed with anti-HA antibody and performed western with anti-Myc antibody to detect co-immunoprecipitated Xbra. (**c**) Schematic diagram of *foxd4l1.1* constructs containing different domains. The three domains were the N-terminal activation domain (“acidic blob”), the winged-helix domain (WHD) and the C-terminal repressor domain (Region-II and P/A/Q). (**e**–**f**) HA-*foxd4l1.1* and Myc-*xbra* were injected. Anti-Myc antibody (Xbra) was used to immunoprecipitate the endogenous *ventx1.1* promoter region. *Ventx1.1* coding region primers used for PCR as a negative control. Fold Enrichment Method used to normalize ChIP-qPCR. All relative promoter activity data are shown as the mean ± SE.

Our previous study reported that XbRE (Xbra response elements) also locates within the *ventx1.1* (− 157) promoter construct (previously designated as *ventx1.1 *(− 103))^15^. XbRE (Xbra) cooperates synergistically with BRE (BMP response element and direct binding site of Smad1, CAGA, − 233 to − 216 region of *ventx1.1* promoter; highlighted in green in Fig. s3) to activate *ventx1.1* transcription^16^. In the present study, we found that FRE1 (ATAAAAA, − 82 to − 76 region of *ventx1.1* promoter) was located within the *ventx1.1* (− 157) in close proximity to XbRE (ATCA*CAC*TT, − 107 to − 99 region of the promoter, Xbra binding site; highlighted in purple in Fig. s3). To examine whether *EnRfoxd4l1.1* inhibits *xbra*-induced activation of *ventx1.1* transcription, v*entx1.1* (− 157) was co-injected with and without *xbra* and *EnRfoxd4l1.1*, in combination or separately. *EnRfoxd4l1.1* abolished *xbra*-induced relative promoter activity of *ventx1.1* (− 157) (Fig. 3a). The physical interaction between Foxd4l1.1 and Xbra was then examined. Embryos were co-injected at one-cell stage with HA-*foxd4l1.1* and Myc-*xbra* mRNA constructs as shown in Fig. 3b,d in different groups. The injected embryos were collected at stage 11.5. Cell lysates were immunoprecipitated with α-HA polyclonal antibody and resolved by electrophoresis in 10% SDS–polyacrylamide gels. Western blotting of Myc-Xbra was performed by using an Myc antibody. Foxd4l1.1 bound to Xbra and the C-terminal of Foxd4l1.1 was required for the physical interaction with Xbra (Fig. 3b,d). To examine whether Xbra still bound within the promoter region of endogenous *ventx1.1* in presence of Foxd4l1.1, Myc-*xbra* was injected with and without HA-*foxd4l1.1* to perform a ChIP-PCR assay. The results indicated that Foxd4l1.1 notably reduced Xbra binding within the endogenous *ventx1.1* promoter (Fig. 3e (ChIP-PCR), f (ChIP-qPCR)). Collectively, the results showed that Foxd4l1.1 binds to Xbra and inhibits the binding of Xbra within the *ventx1.1* promoter, leading to inhibition of *ventx1.1* expression.

### Foxd4l1.1 abolishes Smad1-induced transcription activation of *ventx1.1*

**Figure 4:**
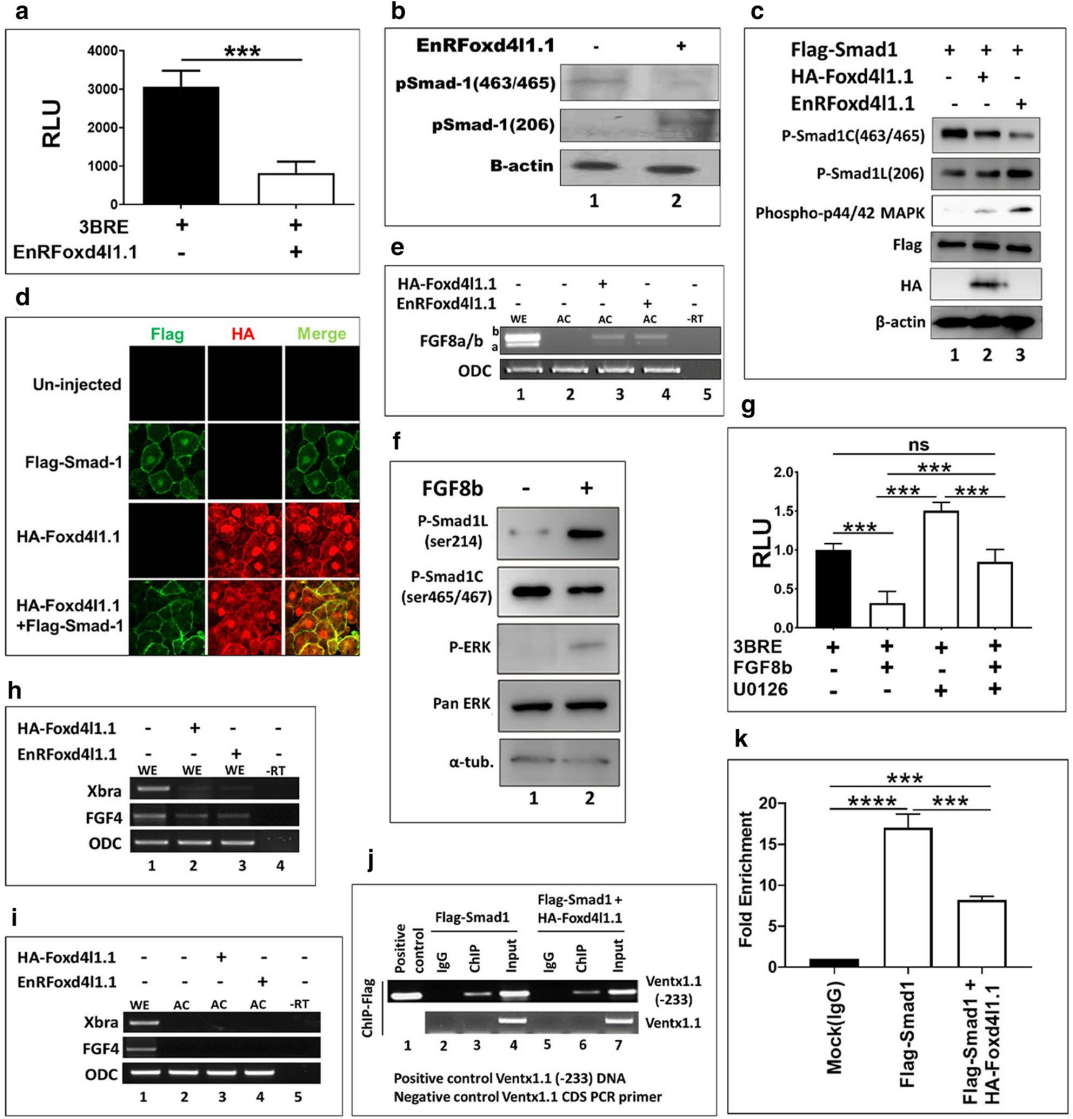
Foxd4l1.1 abolishes Smad1-induced transcription activation of *ventx1.1*. (**a**) Luciferase assays were performed with the injected 3BRE-reporter gene construct with and without *EnRfoxd4l1.1.* (**b**) *EnRfoxd4l1.1* was injected and western blot was performed with anti-Smad1 (phospho S463/S465) and anti-Smad1 (phospho S206) antibodies to detect endogenous Smad1. (**c**) Flag-*smad1* was injected with or without HA-*foxd4l1.1* or *EnRfoxd4l1.1* separately, were analyzed anti-Smad1 (phospho S463/S465), (phospho S206) and phospho-p44/42 MAPK antibodies. (**d**) HA-*foxd4l1.1* and Flag-*smad1* injected separately and together, which were analyzed nuclear localization of Flag-Smad1 by confocal microscopy. (**e**, **h**, **i**) HA-*foxd4l1.1* and *EnRfoxd4l1.1* injected, RT-PCR of *fgf8a/b*, *xbra* and *fgf4* were performed. (**f**) *FGF8b* mRNA was injected and western blot was performed with anti-Smad1 (phospho S463/S465), (phospho S206) antibodies and phospho-p44/42 MAPK antibodies. (**g**) Luciferase assays were performed with the injected 3BRE-reporter gene construct with and without *fgf8b* (treated and untreated with U0126) in different sets. (**j**–**k**) Flag-*smad1* injected with and without HA-*foxd4l1.1* to perform ChIP-PCR assay. Immunoprecipitation performed with Anti-Flag antibody (Smad1). *Ventx1.1* (− 233) promoter DNA was used as a positive control while the *ventx1.1* coding region primers for PCR were used as a negative control for all ChIP experiments. Fold Enrichment Method used to normalize ChIP-qPCR.

*EnRfoxd4l1.1* inhibited expression of BMP4 and its target genes, resulting in the neuroectoderm formation in the animal cap explants (Fig. 1a,b). A previous study demonstrated that ventrally injected *foxd4l1.1* mRNA reduces the number of pSmad1/5/8 positive cells in the ventral epidermis region of embryos^34^ and also inhibits expression of BMP4-targeted genes, *ap2* and *epi-keratin,* while it induces the expression of chordin-stabilizing factor, *sizzled* (*szl*)^34^. In *Xenopus*, the expression domains of *szl* and *bmp4* overlap and it was shown that *szl* expression is strongly dependent on BMP4^35^. Therefore, we examined whether *foxd4l1.1* indeed inhibits Smad1 activity and Smad1-induced transcription activation of *ventx1.1*. To examine the Smad1 activity, 3BRE (triple-repeat of BMP4 response elements of activated Smad1 binding sites, 3XCAGACA^16^) reporter gene construct was injected with and without *EnRfoxd4l1.1* at the one-cell stage and collected the injected embryos at stage 11 for reporter gene assays. *EnRfoxd4l1.1* significantly decreased the relative reporter activity of 3BRE construct compared to that of 3BRE alone (Fig. 4a). Smad1 activity is reportedly positively dependent on its C-terminal phosphorylation and negatively on its linker region phosphorylation^21^. Since the cytoplasmic retention of Smad1 is mediated either by inhibition of its C-terminal phosphorylation (pSer-463/465) (BMP4 inhibition dependent) or the increase of MAPK-mediated linker region phosphorylation (pSer-206) (FGF dependent)^21,22,39,40^, we examined the phosphorylation status of Smad1 to test on which pathway mediated *ventx1.1* repression by *foxd4l1.1*. Our results indicated that *foxd4l1.1* not only reduced C-terminal phosphorylation of endogenous Smad1 (Fig. 4b, first line, pSmad1(463/465)), but it also increased linker region phosphorylation in *Xenopus* embryos (Fig. 4b, 2nd line, pSmad1(206)). We also examined phosphorylation changes for overexpressed Flag-Smad1 and changes in phospho-MAPK levels with and without *EnRfoxd4l1.1* and *foxd4l1.1* expression (Fig. 4c). Flag-Smad1 phosphorylation and phospho-MAPK levels were enhanced in *foxd4l1.1* and *EnRfoxd4l1.1* injected embryos (Fig. 4c). We then examined the localization of Smad1 in presence and absence of Foxd4l1.1 using immunofluorescence staining of animal cap explants. Results showed that Foxd4l1.1 led to cytoplasmic retention of Smad1 with complete exclusion from the nucleus for Smad1 in animal cap explants (Fig. 4d). Figure 4b,c indicated that *foxd4l1.1* increased the linker region phosphorylation of Smad1. We then attempted to address how *foxd4l1.1* could increase FGF/MAPK signaling to catalyze the linker region phosphorylation of Smad1 with the ectopic expression of Foxd4l1.1. *Foxd4l1.1* and *EnRfoxd4l1.1* induced *fgf8a/b* expression in animal cap explants (Fig. 4e). We confirmed whether presence of *fgf8b* affected the linker region phosphorylation of Smad1 and Erk (p44/42 MAPK) phosphorylation in our system (Fig. 4f). We observed the reduction of 3BRE activity by *EnRfoxd4l1.1*. (Fig. 4a). Similarly, the 3BRE activity was reduced in presence of *fgf8b* and the reduction was recovered by the MEK inhibitor, U0126 (Fig. 4g). These results indicated that *foxd4l1.1* not only inhibits Smad1 activation, but may also lead to cytoplasmic retention of Smad1 by activating FGF signaling. Since FGF signaling induces expression of *xbra*, which is a well-known inducer of mesoderm and is neural inhibitory in *Xenopus*^15,24^, we further examined *xbra* and *fgf4 (efgf)* expression with and without *foxd4l1.1* in gastrula embryos and animal cap explants. *Foxd4l1.1* strongly inhibited *xbra* expression in whole embryos (Fig. 4h) and *xbra* and *fgf4* were not expressed in *foxd4l1.1* injected animal caps (Fig. 4i), indicating that Foxd4l1.1 activates FGF/MAPK signaling for Smad1 retention in the cytoplasm (Fig. 4c,d), but inhibits efgf and *xbra* expression (Fig. 4h). We then examined whether Foxd4l1.1 inhibits the direct binding of Smad1 within the proximal region of endogenous *ventx1.1* promoter. The ChIP-PCR results indicated that ectopic expression of HA-Foxd4l1.1 reduced the direct binding of Smad1 within the proximal region of *ventx1.1* promoter (Fig. 4j (ChIP-PCR), 4 k (ChIP-qPCR)). In this study, we did not conclusively show that phosphorylation of Smad1 was likely to be initially inhibited by Foxd4l1.1; however, the results collectively indicated that (1) Foxd4l1.1 activates FGF8/MAPK signaling (but does not increase *xbra* expression) to induce linker region phosphorylation (pSer-206) of Smad1, which leads to cytoplasmic retention of Smad1 and contributes to transcription inhibition of neural repressor, *ventx1.1*. (2) Foxd4l1.1/FGF/MAPK axis mediated Smad1 inactivation may lead to inhibition of zygotic expression of *bmp4*, resulting in reduction of BMP4 levels, leading to reduced C-terminal phosphorylation of Smad1 and its downstream target genes including *ventx1.1.* Taken together, Foxd4l1.1-mediated inhibition of BMP4/Smad1/Ventx1.1 axis may contribute to preventing neuroectoderm to ectoderm transition in dorsal ectoderm (neuroectoderm) region of *Xenopus* embryos.

### Foxd4l1.1 inhibits Xbra-Smad1-induced synergistic activation of *ventx1.1*

**Figure 5:**
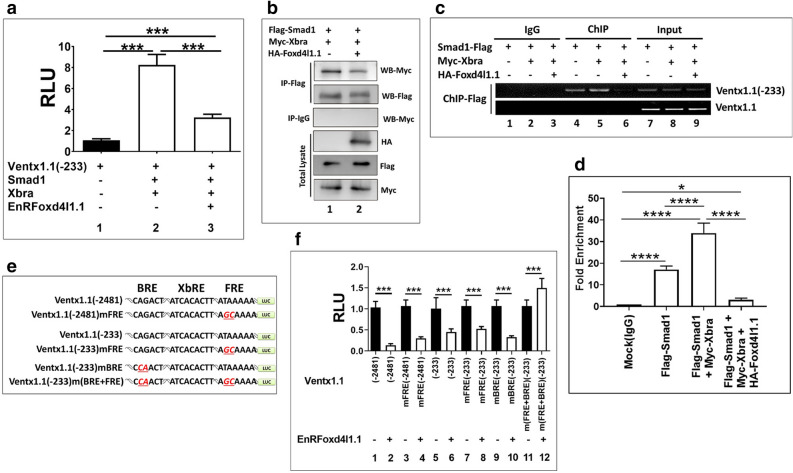
Foxd4l1.1 inhibits Xbra-Smad1-induced synergistic activation of *ventx1.1*. (**a)** Luciferase reporter assay; *ventx1.1* (− 233) promoter construct was injected alone. Additionally, *ventx1.1* (− 233) were co-injected with *smad1*, *xbra* and *EnRfoxd4l1.1.* (**b**) Immunoprecipitation assays were performed to check HA-Foxd4l1.1 effects on the physical interaction of Xbra and Smad1. (**c**, **d**) ChIP-assay performed by anti-Flag antibody (Smad1) and endogenous *ventx1.1* (− 233) was detected by PCR. Fold Enrichment Method used to normalize ChIP-qPCR. (**e**) Site-directed mutagenesis of FRE and BRE in different serially-deleted *ventx1.1* promoter constructs. (**f**) Reporter gene assay of FRE and BRE-mutated different serially-deleted *ventx1.1* promoter constructs with and without *EnRfoxd4l1.1*.

**Figure 6:**
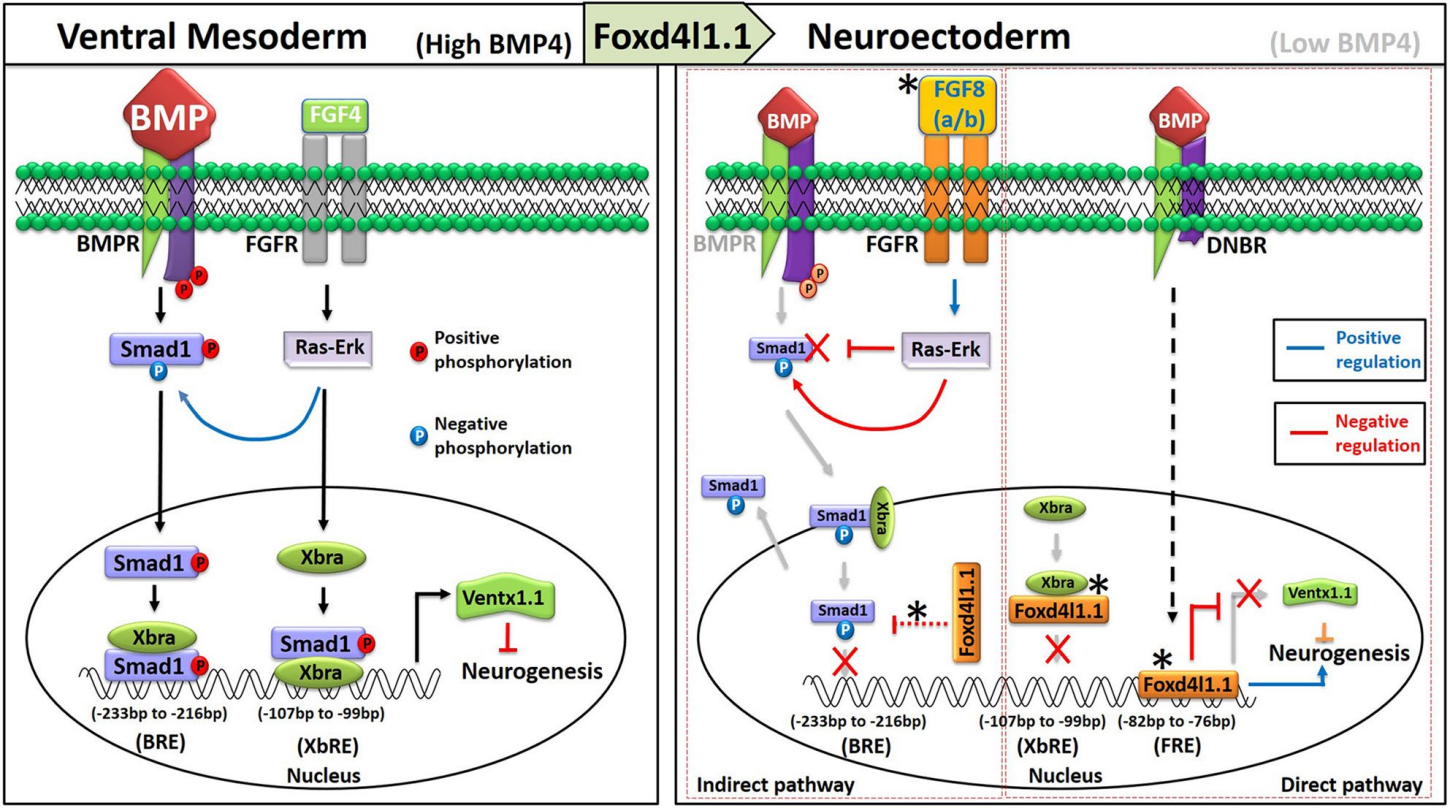
A putative model of Foxd4l1.1-mediated inhibition of *ventx1.1* in its exclusively occupied neuroectoderm regions to trigger neurogenesis in *Xenopus* embryos. A systematic putative model represents Foxd4l1.1-mediated negative regulation of *ventx1.1* transcription during gastrula for neuroectoderm formation in *Xenopus* embryos. In the “neuroectoderm” areas, the BMP/Smad1 levels are relatively low and we propose that the dominant repressory role of Foxd4l1.1 on *ventx1.1* transcription is via the FRE-domain areas bound by Foxd4l1.1. Asterisk marks (*) represent new findings in this study.

The above results showed that Foxd4l1.1 separately inhibits Xbra (Fig. 3a) and Smad1-mediated transcription activation (Fig. 4a) of *ventx1.1*. Foxd4l1.1 physically interacts with Xbra (Fig. 3b–d) and also inhibits activation of Smad1 (Fig. 4b–d). Additionally, ectopic expression of *foxd4l1.1* reduced direct binding of Xbra (Fig. 3e,f) within the *ventx1.1* promoter region. Our previous study has documented that Smad1 and Xbra physically interact in a C-terminal phosphorylation dependent manner for Smad1 in *Xenopus* and that Smad1/Xbra complex synergistically increases *ventx1.1* transcription^15^. Thus, we next inquired whether Foxd4l1.1 inhibits Smad1/Xbra-mediated synergistic activation of *ventx1.1* transcription. We used a *ventx1.1* (− 233) promoter construct which contains all three response elements including BRE, XbRE and FRE1 (Foxd4l1.1 binding site). Results showed that ectopic expression of *EnRfoxd4l1.1* significantly decreased synergistic activation of *ventx1.1* (− 233) promoter activity which was increased by *smad1* and *xbra* in absence of *EnRfoxd4l1.1* (Fig. 5a). *EnRfoxd4l1.1* inhibited synergistic activation of *ventx1.1* transcription, this indicated that repressor activity of Foxd4l1.1 is critical for the inhibition of *ventx1.1* (− 233) promoter activity. Foxd4l1.1 may be inhibiting the physical interaction of Xbra and Smad1. Foxd4l1.1 actually reduced the physical interaction of Xbra and Smad1 (Figs. 5b and s4) and abolished Xbra-induced direct binding of Smad1 within the 5′-flanking proximal region of *ventx1.1* promoter (Fig. 5c,d). *EnRfoxd4l1.1* reduced the relative promoter activity and the relative mRNA level of *ventx1.1* up to eightfold (Fig. 2a), while *EnRfoxd4l1.1* inhibited the relative promoter activity of *ventx1.1* (− 157) construct up to 0.5-fold (Fig. 2c, bars 9–10). These led to the possibility that the *ventx1.1* (− 2481) promoter region may contain more than one consensus cis-acting FRE, jointly involved in negative regulation of *ventx1.1* transcription. Thus, we further mapped the *ventx1.1* (− 2481) promoter region and found 6 more putative cis-acting FRE (RTAAAYA) sites in − 2481 promoter region of *ventx1.1* (Fig. s3). We next mutated the confirmed-FRE1 (− 82 to − 76 region) of *ventx1.1 *(− 2481) promoter construct (Fig. 5e). We co-injected the different FRE1-mutated constructs with and without *EnRfoxd4l1.1* at the one-cell stage. The reporter activities of FRE1-mutated *ventx1.1* (− 2481 and − 233 mFRE1) constructs were reduced by co-injection with *EnRfoxd4l1.1* mRNA albeit the reduction was less than those of wild type *ventx1.1* (− 2481 and − 233 promoter constructs (Fig. 5f, bar2 vs 4 and bar6 vs 8). This indicated that the other putative cis-acting FRE sites (consensus RTAAAYA) in the *ventx1.1* (− 2481) may also contribute to *EnRFoxd4l1.1*-mediated reduction of *ventx1.1* (− 2481) mFRE1 reporter activity. The *EnRFoxd4l1.1*-mediated reduction was recovered by reducing the number of putative FREs in the *ventx1.1* promoter (Fig. 5f, compare the reduction levels of − 2481 in Fig. 5f, bars 1 and 2), − 2481 mFRE (bars 3 and 4), − 233 (bars 5 and 6) and – 233 mFRE (bars 7 and 8)) reporter constructs. As expected, neither FRE-mutated *ventx1.1* (− 233) mFRE nor BRE-mutated *ventx1.1* (− 233) mBRE constructs completely abolished EnRfoxd4l1.1-mediated reduction of the relative promoter activity (Fig. 5f, bars 7 and 8 and bars 9 and 10, respectively). We found that *EnRfoxd4l1.1*-mediated reduction was completely abolished in the doubly-mutated *ventx1.1* (− 233) m(BRE + FRE1) promoter construct (Fig. 5f, bars 11 and 12). *EnRFoxd4l1.1* actually induced the relative promoter activity of doubly-mutated *ventx1.1* (− 233) m(BRE + FRE) construct (Fig. 5f, bars 11 and 12). Overall, *EnRfoxd4l1.1* also inhibited Xbra/Smad1-induced synergistic activation of *ventx1.1* transcription activation (Fig. 5a). Additionally, Foxd4l1.1 reduced the physical interaction between Xbra and Smad1 (Fig. 5b), and Xbra-induced direct binding of Smad1 within the endogenous promoter region of *ventx1.1* (Fig. 5c, lane5 vs lane6). Taken together, we proposed a systematic model of *foxd4l1.1*-mediated transcriptional inhibition of neural repressor *ventx1.1* and inhibition of Xbra/Smad1-induced *ventx1.1* transcription in its exclusively occupied region, leading to neuroectoderm formation in *Xenopus* embryos (represented in Fig. 6, under “neuroectoderm”). This diagram also depicts the reported negative and positive regulation for the BMP4-Smad1-ventx1.1 axis by the FGF-MEK-Erk-Xbra axis in ventral mesoderm (Fig. 6, under “ventral mesoderm”).

## Discussion

In the present study, we wanted to uncover a potential mechanism for non-neural ectoderm exclusion seen in neural ectoderm areas as part of an established transcriptional gene regulatory network (GRN). Here, we focused on elaborating the repressive activity of the neural specific transcription factor (TF), *foxd4l1.1*. *Foxd4l1.1* is one of a number of evolutionally conserved earliest repressor TFs produced in neuroectoderm territory, post inhibition of BMP signaling in the dorsal ectoderm region. We found that *foxd4l1.1,* as a neural ectoderm specific TF, repressed *ventx1.1,* a neural repressor TF. *Ventx1.1* as a neural repressor gene is an immediate early zygotic repressor TF, which is a direct target of BMP/Smad1 and FGF/Xbra signaling in the ventral ectoderm and mesoderm regions^15,16^. Together, our results suggest that repressive non-neural and neural TFs are mutually antagonistic in specifying the non-neural versus neural ectoderm activation areas in the nucleus. The implication and significance of this study is discussed below from the view point of the factors involved in neural induction. The role of extracellular levels of BMP in specifying naive ectoderm cells either to become epidermal or neural ectodermal has been understood since the mid 1990s^5–8^, and that the vertebrate neuroectoderm develops through the inductive signals emanating from the dorsal mesoderm of Spemann organizer has been a finding since 1924^41^. However, only recently, the discovery that the organizer is an antagonist center has led to a shift in thinking of neuroectoderm specification being a default neurogenesis process rather than an active neural induction one^11,42^. Various studies indicate that across vertebrates, including for zebrafish, frog, and mouse embryos, input from BMP4 is required for ectoderm formation and that inhibition of BMP signaling has a conserved role for anterior neuroectoderm formation^27^. BMP4 is among more than 30 known BMP proteins that are mainly involved in epidermal induction and neural inhibition^8,11,13^. BMP signals express target genes that include *gata*, *vent* and *msx* families of TFs. Direct target TFs of BMP signaling that include *gata1b*, *msx1* and *ventx1.1* indeed inhibit neuroectoderm and represent the inhibitory aspect of BMP signals. This indicates that certain TFs in epidermis actively protect against becoming neuroectodermal via a transcriptional repression function of certain direct target TFs of the BMP pathway^12–15^. Albeit which BMP target gene(s) is essential for epidermis specification or the details of the relevant GRNs among various non-neural TFs still remain to be addressed.

In this study, we selected *ventx1.1* as a target TF to examine Foxd4l1.1 function in preventing cells from reverting to a non-neural fate. The reasons on why we focused on *ventx1.1* are the following: First, *ventx1.1* is expressed throughout the embryonic ectoderm in blastula to gastrula embryos such as with *msx1* and *ventx2*. Second, *ventx1.1* induces the epidermis and inhibits the formation of the dorsal mesoderm and neural tissue^14,17^. Third, *ventx1.1* is a neural repressor, being a direct target TF of BMP4^16,18^. Fourth, inhibition of *ventx1.1* induces neural ectoderm together with FGF in animal cap explants, similar to DNBR treated animal caps^8,24^. Fifth, the reporter activity of *ventx1.1* promoter construct is reduced by *foxd4l1.1*^15^. *Ventx1.1* transcript is found in the ventral ectoderm and mesoderm, but is completely absent in the organizer and the neuroectoderm region in early gastrula embryos^43^. Although Ventx1.1 has yet to be proven as an essential master TF among the direct target BMP TFs in epidermal specification, our previous study that *ventx1.1* directly represses *foxd4l1.1* led to examine the reciprocal repression between them in specifying the non-neural versus neural ectoderm fates at the transcription level. For a given ectodermal region, among exclusively expressed neuroectoderm or ectoderm genes, certain TFs may function as repressors in excluding expression of genes of alternate fates. Such a proposal has been put forward by Sasai^25^. Once, the neural ectoderm is formed and neural transcription factors (nTFs) are expressed, some of the earliest expressed nTFs are needed to prevent cells from reverting to a non-neural fate^25,26^. The primary neuroectoderm expresses various transcription factors that may block it from becoming an epidermis^25–28^.

*Foxd4l1.1* (*xfd-12*, *xflip*, *foxd5a*, *b*) transcript is exclusively expressed in the superficial layer, namely neuroectoderm, of cells above the dorsal lip of the Spemann organizer territory at the early gastrula^31,43^. Both Foxd4l1.1 and Ventx1.1 contain a strong repressive domain^17,18,37,44^ with mutually exclusive expression in the ectoderm/ventral mesoderm and the neuroectoderm/organizer, respectively, at the time of the ectoderm/neuroectoderm commitment for early gastrula. We hypothesized that BMP signal modulation leads to expression of non-neural versus neural TFs, with at least one being essential in preventing cells from reverting to alternate fates. We thus examined *foxd4l1.1* as one of the earliest expressed nTFs, which block neuroectoderm from becoming epidermis. Exclusive presence of a repressive gene in a given territory would at least be a strategy to exclude essential gene expression involved in alternate germ layer commitment. For example, ectopic overexpression of organizer genes including *gsc*, *chordin* and *noggin* in ventral region of 4-cell stage embryos leads to two-axis formation in whole embryos. On the other hand, overexpression of ventral specific genes including *vent1.1*, *vent1.2* and *bmp4* in dorsal region of 4-cell stage embryos leads to headless embryos. As such, overexpression studies in developing embryos suggest that ectopic signaling or presence of certain TF(s) in competent cells convert their GRNs.

Reciprocal transcriptional repression of ventral and dorsal opposing homeobox genes *gsc* and *ventx1/2* has also been proposed to in part mediate dorsoventral patterning to ensure robust and reproducible embryonic development through triple depletion of *gsc*, *ventx1*, and *ventx2*^45^. Various TFs have been proposed and examined for being reciprocal means of repression in early vertebrate embryogenesis^46,47^. Involvement of *foxd4l1.1* and *ventx1.1* in neuroectoderm versus ectoderm specification cannot be ruled out in the present study and require further study. However, in this work, we mainly focused on the maintenance/protective role of *foxd4l1.1* in the expressed cells of neuroectoderm for fate reversion.

From our previous work, inhibition of BMP signaling induces *foxd4l1.1* expression, which is directly repressed by Ventx1.1^18^. In the present study, *foxd4l1.1* induced neuroectoderm formation which was also inhibited by co-injection of *ventx1.1* (Fig. 1b), indicating that *ventx1.1* needs to be repressed for proper neuroectoderm formation in both cases of *foxd4l1.1* induced neuroectoderm and DNBR induced *foxd4l1.1* expression. In this study, we show that *foxd4l1.1* inhibited *ventx1.1* expression. We examined whether the inhibition was direct or indirect (or both). It was shown that Foxd4l1.1 directly binds to one of the cis-acting FREs of *ventx1.1* (FRE1; ATAAAA, − 82 to − 76 region of the promoter) to inhibit *ventx1.1* transcription (Fig. 2e–g). We also found an indirect means of *foxd4l1.1* mediated *ventx1.1* repression, separately occurring through Xbra and Smad1. For Xbra inhibition, Foxd4l1.1 bound to Xbra protein to diminish its affinity to the XbRE element (ATCA*CAC*TT, − 107 to − 99 region) of *ventx1.1* promoter (Fig. 3b–f). In addition, *foxd4l1.1* inhibited *xbra* expression (Fig. 4h), although we did not exactly elaborate on how *foxd4l1.1* inhibits *xbra* expression. We speculate that it is from a direct inhibition of *xbra* expression though Foxd4l1.1 binding to the 5′-flanking/promoter region of *xbra*. A genome-wide ChIP-Seq analysis of Foxd4l1.1 implicated such a pathway with Foxd4l1.1 binding to the 5′-flanking region of *xbra* in *Xenopus* embryos (data not shown). However, the exact mechanism remains to be demonstrated.

For *foxd4l1.1* mediated Smad1 inhibition, it has been documented that ventrally injected *foxd4l1.1* reduces the number of phosphorylated Smad1 (activated Smad1) positive cells and inhibits BMP4/Smad1 downstream targets *epi-keratin* and *ap2* in *Xenopus* embryos^34^. Furthermore, *foxd4l1.1* increases expression of *szl*, suggesting that *foxd4l1.1* reduces nuclear localization of Smad1 either by inhibiting *bmp4* expression or by Sizzled-mediated inhibition of BMP4 in a Chordin-dependent manner^34^. In the present study, we found that *foxd4l1.1* reduced *bmp4* expression (Fig. 1a, lane2, 4 vs lane5). Currently, we do not know exactly how *foxd4l1.1* downregulated *bmp4* expression. We and others have elaborated on the possibility of BMP expression being regulated by a positive feedback loop^48,49^. *Foxd4l1.1* mediated reduction of *bmp4* expression may be through inhibiting activation of Smad1 since activated Smad1 may be involved in zygotic *bmp4* expression during mid-blastula transition^50^. We confirmed Smad1 inactivation using a Smad1 specific reporter (3BRE) (Fig. 4a). Interestingly, we found that *foxd4l1.1* increased linker region phosphorylation of Smad1 (pSer-206) (Fig. 4b,c). Since Smad1 is negatively regulated by FGF/MEK/Erk-mediated phosphorylation of Smad1 linker region^21,22^, we elaborated on FGF signal involvement. We observed that both *foxd4l1.1* and *EnRfoxd4l1.1* increased *fgf8a/b* expression (Fig. 4e). These suggested that *foxd4l1.1* increased Smad1 linker region phosphorylation could be mediated by FGF8/MAPK signaling. Our confocal image results showed that *foxd4l1.1* led to a cytoplasmic retention of Smad1 and completely excluded nuclear localization of Smad1 in animal cap explants (Fig. 4d). These observations are basically the same as reported by Yan et al. (2009)^34^. We used animal cap explants, while Yan’s experiments used the ventral epidermal part in whole embryos. A noted difference was the detection of endogenous phospho-Smad1/5/8 in Yan’s report, while, we overexpressed tagged Smad1 and detected the ectopically expressed Smad1 using confocal microscopy. *Foxd4l1.1* increased *fgf8a/b* expression in animal cap explants (Fig. 4e). The question of whether other *fgf*s in addition to *fgf8a/b* are also involved could be raised. We examined the expression of *fgf4 (efgf)* and *xbra* with both *foxd4l1.1* and *EnRfoxd4l1.1* injected embryos. Neither injection induced *xbra* or *fgf4* in the animal cap explants, suggesting that FGF4/MAP-kinase/xbra loop is not involved in *foxd4l1.1* mediated Smad1 linker phosphorylation. Furthermore, whether *fgf8a/b* induction occurs through Foxd4l1.1 activator function or indirectly through Foxd4l1.1 repressor function would be interesting to explore. Both *foxd4l1.1* mRNAs induced *fgf8a/b*, implying that *fgf8a/b* induction may occur indirectly through Foxd4l1.1 repressor function and Xbra/FGF positive feedback loop was not the reason for Smad1 inactivation. Similar to Foxd4l1.1, Zbtb14 reduces the levels of phosphorylated Smad1/5/8 in *Xenopus*^51^. Overexpression of *zbtb14* promotes neural induction similar to that of *foxd4l1.1*. While BMP inhibition and overexpression of *foxd4l1.1* induces anterior neural tissue, *zbtb14* promotes posterior neural tissue and suppresses anterior neural tissue. Presently, we cannot delineate the connection between Foxd4l1.1 and Zbtb14 in neural induction and Smad1 inactivation, and details of *foxd4l1.1* mediated BMP/Smad1 signal attenuation and *fgf8* involvement during neuroectoderm formation await more elaboration.

Previously, our studies show that Smad1 and Xbra physically interact and synergistically cooperate to increase *ventx1.1* transcription in *Xenopus* embryos^15^. Foxd4l1.1 reduced Smad1 and Xbra physical interaction possibly, first, due to reduced C-terminal phosphorylation of Smad1 via reduced BMP expression, and second, via competition for Xbra since both Foxd4l1.1 and Smad1 bind to Xbra protein (Figs. 5b and s4). However, the latter reason is less essential since both Foxd4l1.1 and EnRFoxd4l1.1 (which does not contain Xbra binding C-terminal domain) reduced Smad1 and Xbra physical interaction (Fig. 5b, Fig. s4a,b). C-terminal phosphorylation of Smad1 is crucially required for the interaction with the N-terminal domain of Xbra^52^. However, this explanation is not enough to address more effective Foxd4l1.1 mediated reduction of Smad1 binding on the *ventx1.1* promoter with presence of Xbra (Fig. 5c,d (lane 5 vs lane6)) when compared with absence of Xbra (Fig. 4j,f). At this moment, we only speculate that the difference may be dependent on the difference of involved FGF characters and additional work is necessary to explain how Xbra contribute positively and negatively for Smad1 binding on context dependent manner.

*EnRfoxd4l1.1* inhibited the relative promoter activity for *ventx1.1* (− 2481) promoter construct by up to eightfold while it reduced that of *ventx1.1* (− 157) construct by up to 0.5-fold. This may be due to *ventx1.1* (− 2481) promoter containing more than one functional FRE for Foxd4l1.1. The *ventx1.1* (− 2481) promoter region contains an additional six putative cis-acting FREs for Foxd4l1.1 (Fig. s3, putative FREs; highlighted in red). A point mutation within the confirmed FRE1 in *ventx1.1* (− 2481, − 233 and – 233 mBRE) promoter constructs indicated that the 5′-flanking region of *ventx1.1* contains more than one consensus FRE, actively participating in Foxd4l1.1-mediated negative regulation of *ventx1.1* transcription (Fig. 5f). *EnRFoxd4l1.1* more efficiently inhibited BRE-mutated *ventx1.1* (− 233) mBRE promoter constructs (Fig. 5e,f, bar 9 vs bar 10). Surprisingly, *EnRFoxd4l1.1* increased the relative promoter activity of doubly-mutated *ventx1.1* (− 233) m(BRE + FRE) promoter construct (Fig. 5f bar 11 vs bar12). This induction may be caused by inhibition of endogenous *goosecoid* (*gsc*) expression by *EnRFoxd4l1.1*. Our study demonstrates that *gsc* inhibited *ventx1.1* expression as well as the relative activity of *ventx1.1* promoter construct^16^. *Ventx1.1* (− 233) promoter contained the direct binding response element for Gsc (GRE: ATTTGC, − 195 to − 190 region of the promoter; highlighted in blue in Fig. s3, unpublished data), which was experimentally identified.

In summary, we propose a model for Foxd4l1.1 inhibiting the transcription of *ventx1.1* in its exclusively occupied region of neuroectoderm via the mechanisms elaborated in this work and leading to primary neurogenesis in *Xenopus* embryos (Fig. 6; right panel; highlighted as red and blue lines for the evidence shown in this paper). In this paper, we provide evidence on how Foxd4l1.1 represses *ventx1.1* transcription in neuroectoderm. This work provides an insight on how Foxd4l1.1 negatively regulates the neural repressive BMP-Smad1-ventx axis, specifically at the transcription level for the neural repressor *ventx1.1* and the Foxd4l1.1 exclusively occupied territory of neuroectoderm. In the “neuroectoderm” areas, we propose that the dominant repressory role of Foxd4l1.1 on *ventx1.1* transcription is via the FRE-domain areas bound by Foxd4l1.1 as the BMP/Smad1 levels are relatively low already. In Fig. 6, we depict the direct or indirect regulatory axes, as supported by our data. With the role of extracellular BMP/BMPR in fate determination being in the literature for many years, the intracellular details in transcription regulation of neural/non-neural TFs have been lacking and that this model serves to fill in some of the current gaps in the literature. Homologues of *Xenopus foxd4l1.1* are highly conserved across vertebrates that include zebrafish, mouse and also humans. All the homologues of *foxd4l1.1* are similarly expressed in the neural ectoderm of embryos^53–55^. Recently, Sherman et al. reported that *foxd4* in mouse is required for transition of a pluripotent ES cell to a neuroectodermal stem cell, suggesting that mouse *foxd4* has a similar function to its *Xenopus* orthologue^56^. Similarly, our study may provide an additional insight on neuroectoderm differentiation in early embryogenesis across vertebrates.

## Materials and methods

### Ethics statement

This animal study was conducted in accordance with the regulations of the Institutional Animal Care and Use Committee (IACUC) of Hallym University (Hallym 2012-76, 2013-130, 2019-79). All the research members attended both the educational and training courses for the appropriate care and use of experimental animals at our institution in order to receive an animal use permit. Adult *X. laevis* were grown in approved containers by authorized personnel for laboratory animal maintenance, at a 12 h light/dark (LD 12:12 h) cycle and at 18℃ according to the guidelines of the Institute of Laboratory Animal Resources.

### DNA and RNA preparation

All mRNA used for this study were synthesized from linearized vectors. All vectors were linearized with the appropriate restriction enzymes, including *EnRfoxd4l1.1*: T7, BamHI, Myc-*xbra*: Sp6, Asp718, HA-*foxd4l1.1*: Sp6, Asp718, and Flag-*smad1*: Sp6, SalI. In vitro transcription was performed by using a MEGAscript kit accordingly to the manufacturer’s instructions (Ambion, Austin, TX). All synthesized mRNAs were quantified by a spectrophotometer at 260/280 nm (SPECTRA max, Molecular Devices, San Jose, CA).

### Cloning of *ventx1.1* (*Xvent-1b, PV.1*) genomic DNA (gDNA)

The cloning of *ventx1.1* gDNA of Chr-3S was performed into the pBluescript SK(-) plasmid (Stratagene, Cedar Creek, TX) as described by Lee et al.^16^.

### *Ventx1.1* promoter constructs

The 2.5 kbs of 5′-flanking region of positive clone was subcloned into the pGL-2 basic plasmid (Promega, Madison, WI) and was designated as the − 2481 bps construct. Serially-deleted *ventx1.1* promoter mutants and triple-repeated BMP4-response element (BRE) were generated from − 2481 bps construct and subcloned into a pGL-2 basic plasmid by PCR amplification^16^. Numbering of the reporter construct was for the distance 5′-upstream of the translation start site (ATG).

### Embryo injection and explants culture

*Xenopus laevis* adults were obtained from the Korean *Xenopus* Resource Center for Research (Seoul, Korea)*. Xenopus* embryos were obtained by in vitro fertilization after induction of female frogs with 500 units of human chorionic gonadotropin (Sigma, St. Louis, MO). RNAs were injected into the animal pole of 1–2 cell stage embryos; animal caps explants were dissected from injected embryos at stage 7–8 and incubated to stage 11 and 24 in L-15 medium for RT-PCR experiments.

### RNA isolation and RT-PCR

*EnRfoxd4l1.1* (280 pg/embryos) and other mRNA (Myc-*xbra* (1 ng/embryo), Flag-*smad1* (1 ng/embryo) and HA-*foxd4l1.1* (3 ng/embryo)) was injected into the animal pole of one-cell stage embryos that were then cultured in 30% MMR solution until stage 8 and also for control non-injected embryos. Animal caps were then dissected from the injected and non-injected embryos and incubated until stage 11 and 24 in L-15 medium. Total RNA was isolated from whole embryos or animal caps using RNA-bee reagent following the manufacturer’s instructions (TEL-TEST, Friendwood, TX) as described by Kumar et al. (2018)^15^. PCR was performed by using oligonucleotides according to the following conditions as described in Table 1.

**Table 1:** List of primers used for PCR amplification.

Gene Name	Sequence	Annealing temp ( °C)	Cycles
*bmp4*	F-5′-GCATGTACGGATAAGTCGATC-3′ R-5′-GATCTCAGACTCAACGGCAC-3′	57	25
*ventx1.1*	F-5′-CCTTCAGCATGGTTCAACAG-3′ R-5′-CATCCTTCTTCCTTGGCATCTCCT-3′	57	26
*ventx1.2*	F-5′-TTCCCTTCAGCATGGTTCAAC-3′ R-5′-GCATCTCCTTGGCATATTTGG-3′	57	25
*ventx2.1*	F-5′- CTACAGCACTAGCACTGACTCAGG-3′ R-5′-TTGGACTGCATGCTGCAATACAGG-3′	57	25
*ngnr*	F-5′-GGATGGTGCTGCTACCGTGCGAGTACC-3′ R-5′-CAAGCGCAGAGTTCAGGTTGTGCATGC-3′	65	25
*ncam*	F-5′-CACAGTTCCACCAAATGC-3′ R-5′-GGAATCAAGCGGTACAGA-3′	57	29
*otx2*	F-5′- GGATGGATTTGTTGCACCAGTC-3′ R-5′- CACTCTCCCAGCTCACTTCTC-3′	57	27
*krox20*	F-5′-AACCGCCCCAGTAAGACC-3′ R-5′-GTGTCAGCCTGTCCTGTTAG-3′	57	32
*hoxB9*	F-5′-TACTTACGGGCTTGGCTGGA-3′ R-5′-AGCGTGTAACCAGTTGGCTG-3′	56	26
*odc*	F-5′-CCCTATAAGACAAGGAATAC-3′ R-5′-TCCATTCCGCTCTCCTGAGCAC-3′	55	25

### Quantitative RT-PCR (qPCR)

The qPCR reactions were performed by using an Applied Biosystems StepOnePlus Real-Time PCR System with KAPA SYBER FAST qPCR Master Mix. All the real-time values were averaged and compared using the threshold cycle (CT) method, in which the amount of target RNA (2 − ΔΔCT) was normalized against the endogenous expression of ODC (ornithine decarboxylase) (ΔCT). The qPCR reactions were performed when RT-PCR reaction results need to be quantified (Fig. s1). The confirmed data as elsewhere published data (vent1.1)^57^ was not repeated. All qPCR reactions were repeated three time using independent samples to present data with standard deviations and statistical significance.

### Luciferase assays

Levels of relative luciferase activity were measured as described by Kumar et al. (2018)^15^.

### Identification of binding sites of transcription factors and site-directed mutagenesis

The binding sites of transcription factors including Foxd4l1.1 (FRE1) and Smad1 (BRE) were identified using serially-deleted reporter gene constructs (Table 2) and site-directed mutagenesis. Site-directed mutagenesis of FRE1 and BRE within ventx1.1 promoter constructs were performed by a site-directed mutagenesis kit (Muta-Direct, iNtRON Biotechnology, Seongnam, Korea) by using the oligonucleotides listed in Table 3. Site-directed mutagenesis of XbRE has been described by Kumar et al.^15^.

**Table 2:** List of primers used for serially-deleted reporter gene constructs.

	Primer name	Sequences (5′ → 3′)
Upstream primers	− 2481	AGTCCTCGAGTACCTGCAACTTACTCGC
	− 419	AGTCCTCGAGCCAACATAAAAGGATAAAGG
	− 233	AGTCCTCGAGACTAACCTGACAGACTCACTGG
	− 216	AGTCCTCGAGACTGGAGCCAGGACCAGG
	− 190	AGTCCTCGAGCTACAAGTGAGAACATAA
	− 157	AGTCCTCGAGTAGCCCATTCTGATAGCC
Downstream primer	− 22	AGTCAAGCTTGATGGAGCCGCTGGAGTTGTG

**Table 3:** List of primers used for site-directed mutagenesis.

Mutated sites	Name	Primer name	Sequences (5′ → 3′)
FRE	mFRE	MT-F F-D5	F-ATATCCATCAAGCTA***GC***AAAACAGAGGCTCAG
		MT-R F-D5	R-CTGAGCCTCTGTTTT***GC***TAGCTTGATGGATAT
BRE	− 233MT	mBRE-F	F-AGTCCTCGAGACTAACCTGAC***CA***ACTCACTGG
		mBRE-R	R-CCAGTGA***GT***TGGTCAGGTTAGTCTCGAGGACT

### Western blotting

The mRNAs were injected at the one-cell stage of embryos and collected at stage 11 for western blots. Non-injected embryos served as the negative control. Collected embryos were lysed in lysis buffer with phosphatase and protease inhibitors, in preparation for resolving the proteins with 10% SDS-PAGE and transfer to a PVDF membrane, with the membrane blocked and incubated with either pSmad1 pSer-463/465 (CS-9511S) or pSmad1 pSer-206 (CS-9553P) antibodies. Following washes of the PVDF membrane, it was incubated with the enzyme-labeled secondary antibody (ADI-SAB-300, Enzo Biochem, Farmingdale, NY). The protein signals were visualized by an ECL detection kit (GE Healthcare, Chicago, IL).

### Immunoprecipitation

Embryos were co-injected at one-cell stage with Flag-*smad1*, HA-*foxd4l1.1* and Myc-*xbra* mRNA constructs in four different groups. The injected embryos were collected at stage 11.5. They were then homogenized in lysis IP buffer. The composition of the IP buffer is described in Kumar et al. (2018)^15^. Cell lysates were cleared by centrifugation and were then incubated with Flag-Smad1 (F-2574, Sigma) monoclonal antibody and α-HA (SC-805, Santa Cruz Biotechnology) polyclonal antibody overnight at 4 °C with the immunocomplexes precipitated by protein A/G beads (SC-2003, Santa Cruz Biotechnology). Proper amounts of precipitated beads-protein complex were boiled in the sample buffer, and resolved by electrophoresis in 10% SDS–polyacrylamide gels. Western blotting of Myc-Xbra (SC-789, Santa Cruz Biotechnology) was performed by using an anti-Myc and secondary antibody anti-mouse (SAB-100, Stressgen, Victoria, BC). Immune complexes were visualized by using an ECL detection kit (GE Healthcare).

### Immunofluorescence

Embryos were injected at one-cell stage into animal pole with mRNAs. Injected and un-injected embryos were dissected at stage 9.5 to perform immunofluorescence as described in Lee et al. (2018)^58^. Dissected animal caps were fixed in 4% paraformaldehyde for 2 h and incubated in PBSTB (0.5% Triton-X, 2% BSA in PBS) for 1 h. The animal caps were incubated with primary antibody for overnight at 4 °C and washed with PBS. The animal caps then were incubated with fluorescent secondary antibodies for 2 h at room temperature. Mounted animal cap images were then obtained and analyzed by confocal microscopy (CLSM II, Carl Zeiss LSM-710) (Carl Zeiss, Oberkochen, Germany) at a magnification of 63X.

### Chromatin immunoprecipitation (ChIP)

Chromatin immunoprecipitation assay was performed as described in Blythe et al. (2009)^59^ Embryos were injected at one-cell stage with mRNA encoding HA-*foxd4l1.1*, Myc-*xbra* and Flag-*smad1* (1 ng/embryo) either separately or in combination. The antibodies used to immunoprecipitate chromatin were anti-HA (SC-805, Santa Cruz Biotechnology), anti-Myc (SC-789, Santa Cruz Biotechnology) polyclonal antibody and anti-Flag (F-2574, Sigma) monoclonal antibody. Normal rabbit IgG (SC-2027, Santa Cruz Biotechnology) and normal mouse IgG (SC-2025, Santa Cruz Biotechnology) used as a negative control. PCR were performed with immunoprecipitated fragmented chromatin using *ventx1.1* (− 233 and − 157) promoter region primers. The Fold Enrichment Method was used to analyze ChIP-qPCR values^60^. “Fold enrichment” is by calculating the ΔCT for the difference between CT values for the ChIP samples using the antibody of interest and the negative control antibody. The primer sequences are shown in Tables 2 and 1.

### Nucleotide sequence accession number

The *ventx1.1* (accession number; AF133122) cDNA sequence has been submitted to GenBank^16^.

### Statistical analysis

Data were analyzed by GraphPad Prism5 (GraphPad, San Diego, CA). Statistical analysis was established using one-way ANOVA. *p* < 0.05 was considered to be significant for difference. Notations: **p ≤ 0.01; ***p ≤ 0.001; n.s., not significant.

## Supplementary information

Supplementary information
