# Supplementary material for: Foxd4l1.1 negatively regulates transcription of neural repressor ventx1.1 during neuroectoderm formation in Xenopus embryos
Source: Sci Rep. 2020 Oct 8;10:16780. doi: 10.1038/s41598-020-73662-4 (PMC7545198; doi:10.1038/s41598-020-73662-4)
Supplement: Supplementary file 1 — Supplementary information [file 41598_2020_73662_MOESM1_ESM.pdf]

Foxd4l1.1 negatively regulates transcription of neural repressor *ventx1.1* during neuroectoderm formation in *Xenopus* embryos

Shiv Kumar[1], Zobia Umair[1]*, Vijay Kumar[1], Santosh Kumar[1], Unjoo Lee[2]**, and Jaebong Kim[1]***

[1]Department of Biochemistry, Institute of Cell Differentiation and Aging, College of Medicine, Hallym University, Chuncheon, Gangwon-Do, 24252, Republic of Korea

[2]Department of Electrical Engineering, Hallym University, Chuncheon, Gangwon-Do, 24252, Republic of Korea

*These authors contributed equally

****Address correspondence to:** [1]Department of Biochemistry, Institute of Cell Differentiation and Aging, College of Medicine, Hallym University, Chuncheon, Gangwon-Do, 24252, Republic of Korea. Fax: +82-33-244-8425; Tel: +82-33-248-2544; E-mail: jbkim@hallym.ac.kr

[2]Department of Electrical Engineering, Hallym University, Chuncheon, Gangwon-Do, 24252, Republic of Korea. Tel: +82-33-248-2354; E-mail: ejlee@hallym.ac.kr

E-mail addresses

- ✓ Shiv Kumar (ars198922@gmail.com)
- ✓ Zobia Umair (zobia.umair@hallym.ac.kr)
- ✓ Vijay Kumar (vijay10187@gmail.com)
- ✓ Santosh Kumar (santosh85aiims@gmail.com)
- ✓ Unjoo Lee (ejlee@hallym.ac.kr)

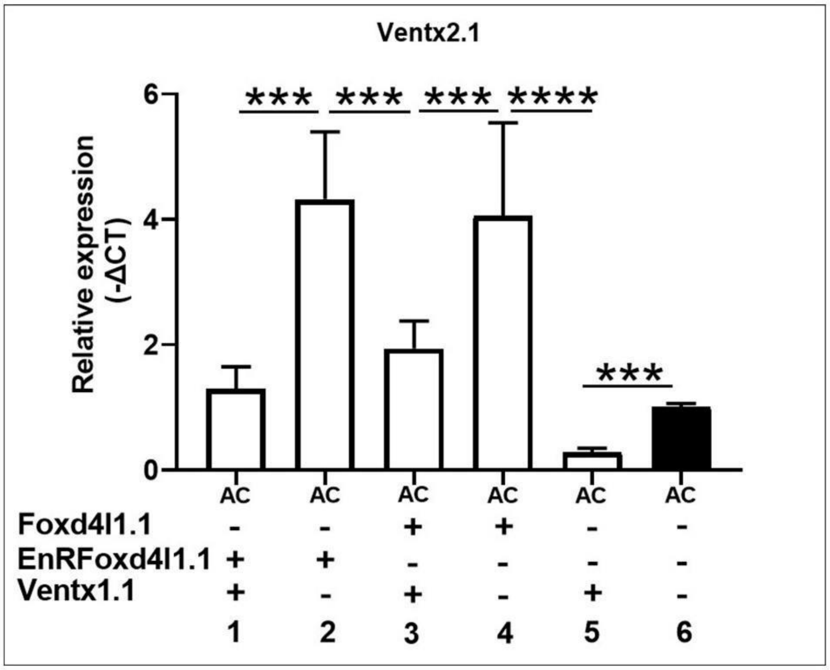

Supplementary Figure 1. Ectopic expression of Foxd4l1.1 regulates *ventx2.1* transcription in animal cap explants of *Xenopus*. EnRFoxd4l1.1 (280 pg/embryos) and HA- Foxd4l1.1 (3ng/embryos) injected at the one-cell stage and dissected the animal cap at stage 8 to grow until stage 11. The expression profiles of ***ventx2.1*** was analyzed by q-PCR. The amount of target gene (2-Δ ΔCT) was normalized against endogenous expression of ODC (ΔCT).

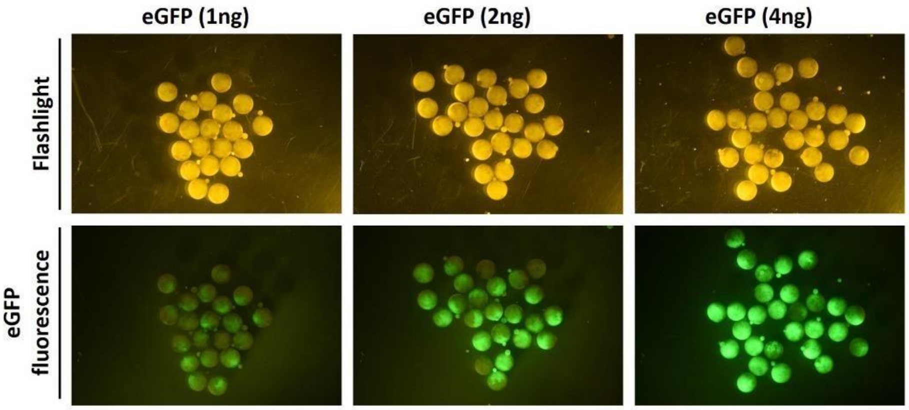

Supplementary Figure 2. Identification of eGFP expression by fluorescence in dose dependent manner. Ventx1.1 (-2481) promoter coinjected with eGFP mRNA (1, 2 and 4 ng/embryos) as control, eGFP fluorescence check at stage 11.

GAATTCAATTGCTTCATTATACATTTTACAAAGGGACTAAGTTTTACCTGCAACTTACTCGCTGCTTTCACAGTAAAACTCCCAAACTTGGCTGCCCTTTTATTA
GACACCAGTGGGATCACCTGACTATAGCTGGAAGCTGTAGTTGGCCATCTTAAATATATTGCAATATATGGACAAATAATCCCTGTTTTGTTTAAAGGGTAAGG
CATTTTTTAGTAGCTGTATGTACAAAATGTCTCAATGTCTTAAATATATTAATAATGGGTTGAGTGCAGAGGATCAAGAGCATTGTCCTATTGCAACTGCAATGA
CACTGAAATGATGA**ATAAAA**ATGCAGCATTGTGGGATAGCGCTAGAAATCTGCCGCGAGGAGTAAGTAAAAAAATCGGGGCATTTGCCCGGGGTAGCAA
GTAGTCTGGGGGAGGGTCTAGGGGGGGTAGGGTATTTTTAAAGTATGGGTTTAGATCTCCTTAAAGGCCAGTGCCACATTTAGGGGGTTAATTATCAAAATC
CAAATTCATCTCATTATTTCTAAAAATATACTCCGACCAAATCCGCATGGGTTATTTCCCCTTATTTATCA**ATAAAA**ACTTGAAAAAAATTGTGAAAATCGTATA
TAAATCAAATCATACTAATTTTTTGGATTTTCTGAATTTCTGTTTTTTTTCAAATTTTTACCCAAAAACCACAAAATCTTCTGATTCCCAAAAAAATTCAACATTTGG
AAAAAACTTGAAAAAAATTGTGAAAATCATACATAAATCAAATCATACAAATTTTTTGGAATTTCCAATTTTCTGATTTTTTTCAAATTTTTACACAAAAACCACAA
CATTTTCTGATTATAGCACGAAATCCAACGCAGGTCAGGATATCCTCAGGACTTCTCTCATTGACTTATATGCAACCTCGGCAGGTCTGAGATGAGGGATTTT
CGGATTCTGACTTTTTCCATTCTTGGGGTATAATAAATCCTGAAAAATTCGAGGTTTTTTTTCCACTAAAAATTCGGATTTTATAGTAAAAAAAAAAATAGAAGTTT
TTGGCATTCTGACTAATAAATAACCCCCTAAGTGTTTCCTGCACTGCCACATATGCACCAGGGG**ATAAAA**GCAGGCCCTGACTGGCAATCTGTGCCAGAG
GGGCTGCTATAAGGTGCCATAGAAAGTCAGTATTTAGTGGGCTGGTAGGCTGGCTGTTTGGCCCTCTGTGTGGGCTGATTGGGCCTCTGTGTACCTGAAAT
GCCAGGGCCTATTTTGAGTCTCAGTCCATACCTAA**ATAAAA**TCGACCAATCCTGTCCAGCCTCTCTTGTGTATACACTGACAGGCACAGGATTAGTCTTTT
TGCCTCCAATAAGGATTAATTATATCTTAGTTGAGATCAAGTACAGGCTACTGTTTTATTATTGCAGAGAAAAAATGATTTGATTATTATGGGGTCTATGGGAGAT
GGTCTTTCCATAATTCAGAACGTGTGGATAACAGGTTTCCGGACCATACCTGTATCTGTACAACAAAACAATAATTTAATAGAACAAACAATACAATAATAAAGC
ACAGTTACAGAAAAGATTAGCTTCTAGGTGTTAAAAAGATGAGTAGAAGGAGGTTCCTGCCAATGCAATGGTTCCAGGCACAGGCAAGGAAAAAGGCTGAT
CCCAGCATAGGGACCTATTCTCAGCCAGCGTTTTTTCGTGAGTGAGAATGAGCCCCTTGGCTTTAGCTTAGGGTAAGGTCACATGGGCAGATTCGTGGAGA
TTAGTTGCCTCACGAGGAAACTGCAGGCAACTTCGGAAAACGAAGCGCCACGAGTGCCATCCTGCTGGCGATTTTTCATTCTAGCTGGCTGGAAGGTAGG
GGAAGGCAGTTCGGGGAAATTGTTGCCCCAAAGAAGAGGAGATTTGTCGCTGGGGCAACTAATCTGCCCTTACCCTTAAAGCACACACAACATCAGCCCT
GTATGACATTCCCAAAGAAGTGTTGTGGCTGCTCCCCATTGTCAGTAATGTGACTTATCCTAAATTTAACCAACCCATGAGACTGGTGCCTGAATTTGGGGAA
AAGTTGCGGAGGCAAAATGCCAGTCTCCTGGTGTGACTTTACTCCAAC**ATAAAA**GGATAAAGGAAAAGAGGTTGTTCTTATTGGTGTGCTCAATAACAAC
ATCAAGGAATGAAAACAAACCTACATTATCTCTTTCCATTGTGCATTTCTCTGCTGTCTGTCCATGGGATTCTGTGCCGGCCAATGCTAATGCCTCCAACTGA
AACCTCCAATATCACAAGGTGAAATCACTAACCTGA**CAGACT**CACTGGAGCCAGGACCAGGGGC**ATTTGC**TACAAGTGAGAAC**ATAAAA**TGCTTACG
TTAATTAGCCCATTCTGATAGCCATTATCCATTTCTGGTTCTCCCTTTGATGTGG**ATCACACTT**GAATATCCATCAAGCT**ATAAAA**ACAGAGGCTCAGCA
GGCCATAGG**CTTTCT**GTACACACAACTCCAGCGGCTCCATCCAGAGCAGGCTTCCCTTCAGC**ATG**

	Site	Sequenses	Location(from ATG)	Remarks
1	**FRE1**	ATAAAA	-82 to -76	Highlighted, **black bold**
2	**XbRE**	ATCACACTT	-107 to -97	Highlighted, **purple bold**
3	**GRE**	ATTTGC	-195 to -190	Highlighted, **blue bold**
4	**BRE**	CAGACT	-223 to -218	Highlighted, **green bold**
5	**FRE**	ATAAAA	-176 to -170	Highlighted, **red bold**
6	**FRE**	ATAAAA	-414 to -408	Highlighted, **red bold**
7	**FRE**	ATAAAA	-1244 to -1238	Highlighted, **red bold**
8	**FRE**	ATAAAA	-1416 to -1410	Highlighted, **red bold**
9	**FRE**	ATAAAA	-1937 to -1931	Highlighted, **red bold**
10	**FRE**	ATAAAA	-2195 to 2189	Highlighted, **red bold**
11	**TSS**	CTTTCT	Start from -53	Arrow (⟶), blue underlined
12	**TLS**	ATG	Start from +1	Red underlined

FRE1, Foxdl1.1 response elements. **XbRE**, Xbra response elements. **GRE**, Gsc response elements.
BRE, BMP4 response elements. **FREs**, Forkhead response elements
TSS, Transcription start site. **TLS**, Translation start site

Supplementary Figure 3. Ventx1.1 promoter (-2481 bp) contains cis-acting elements for several transcription factors including Foxd4l1.1, BMP4, Gsc and Xbra. Upper panel; Ventx1.1 promoter sequence (-2481bp), cis-acting elements highlighted. Lower panel; in table detailed of different sites and position.

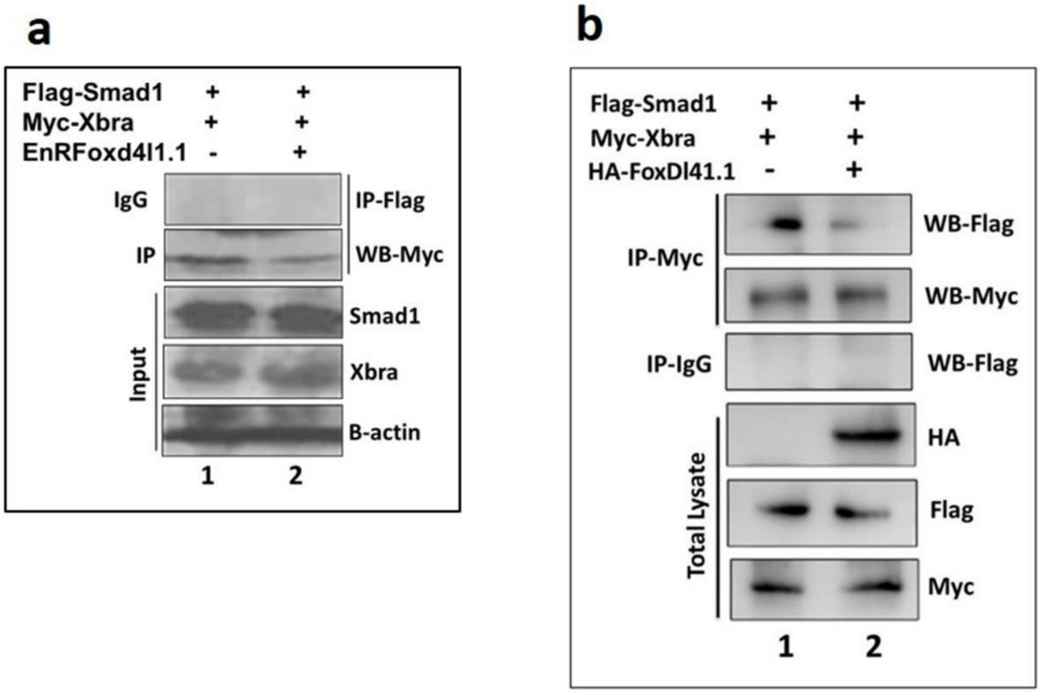

Supplementary Figure 4. Foxd4l1.1 inhibits Xbra-Smad1-induced synergistic activation of ventx1.1. (a-b) Anti-Flag and Anti-Myc Immunoprecipitation was performed to check on EnRFoxd4l1.1 and HA-Foxd4l1.1 effects for the physical interaction of Xbra and Smad1.

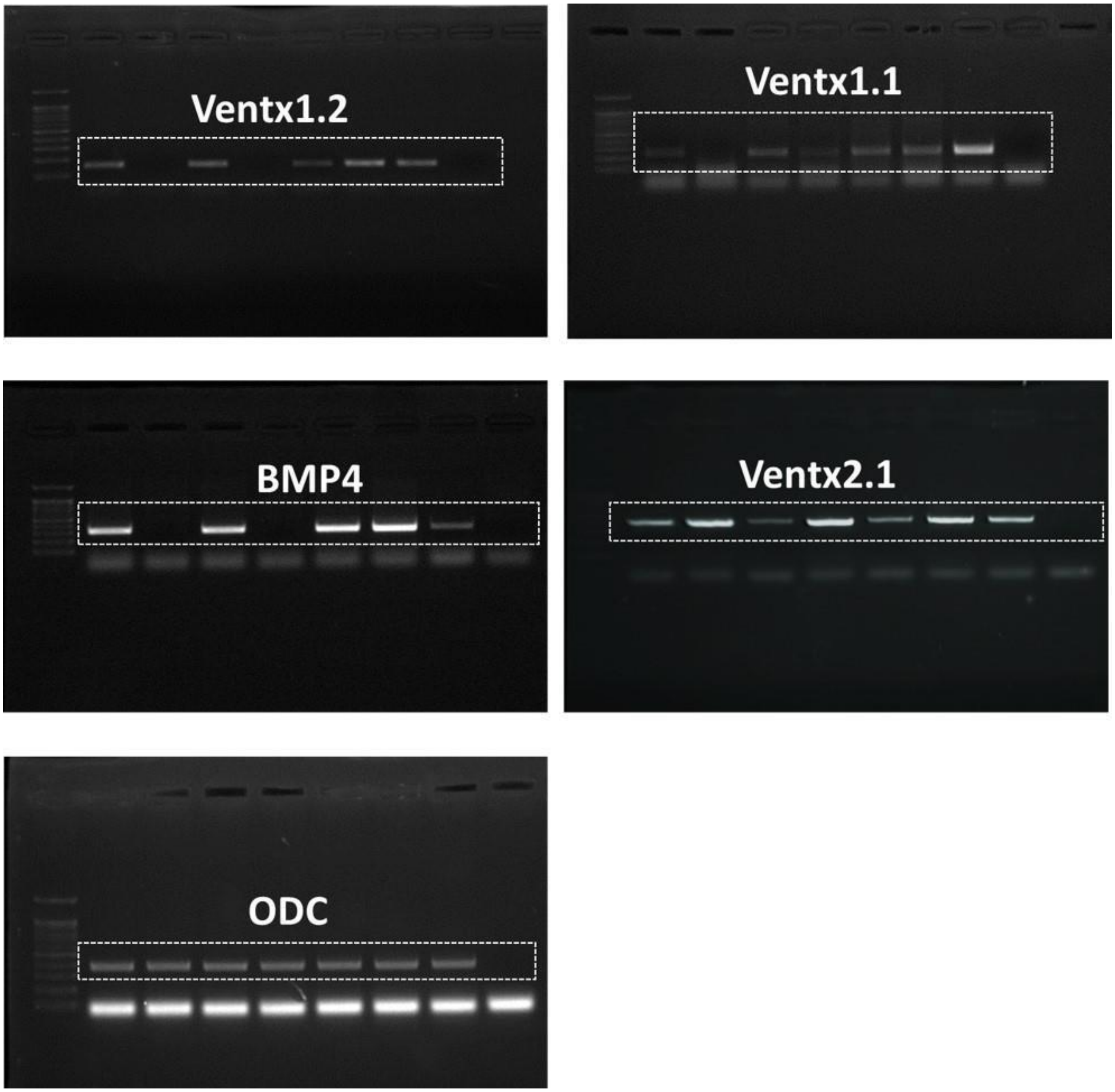

Figure 1a: Full gel images (Dashed boxes indicate the portion of gels included in the figures)

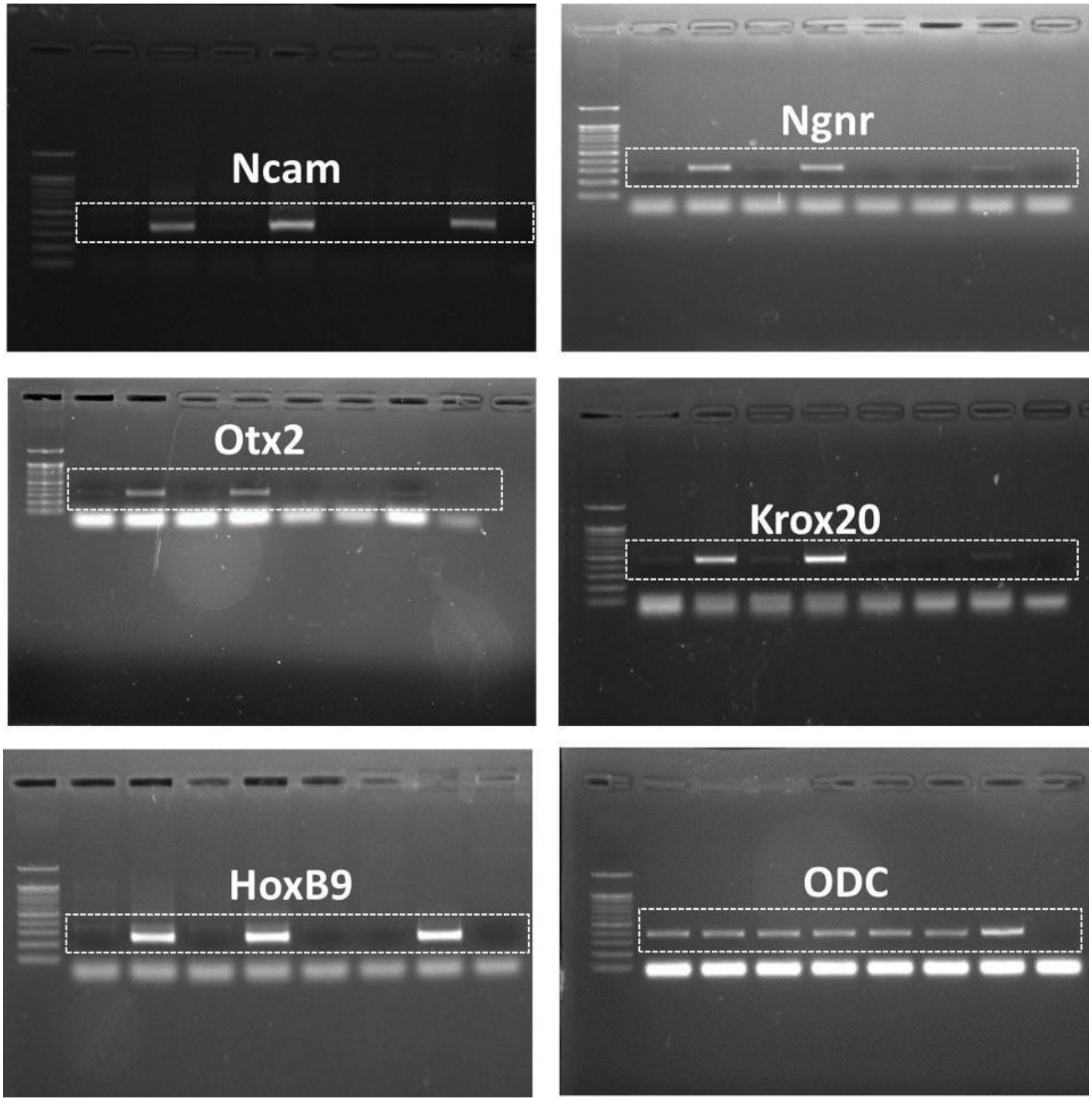

Figure 1b: Full gel images (Dashed boxes indicate the portion of gels included in the figures)

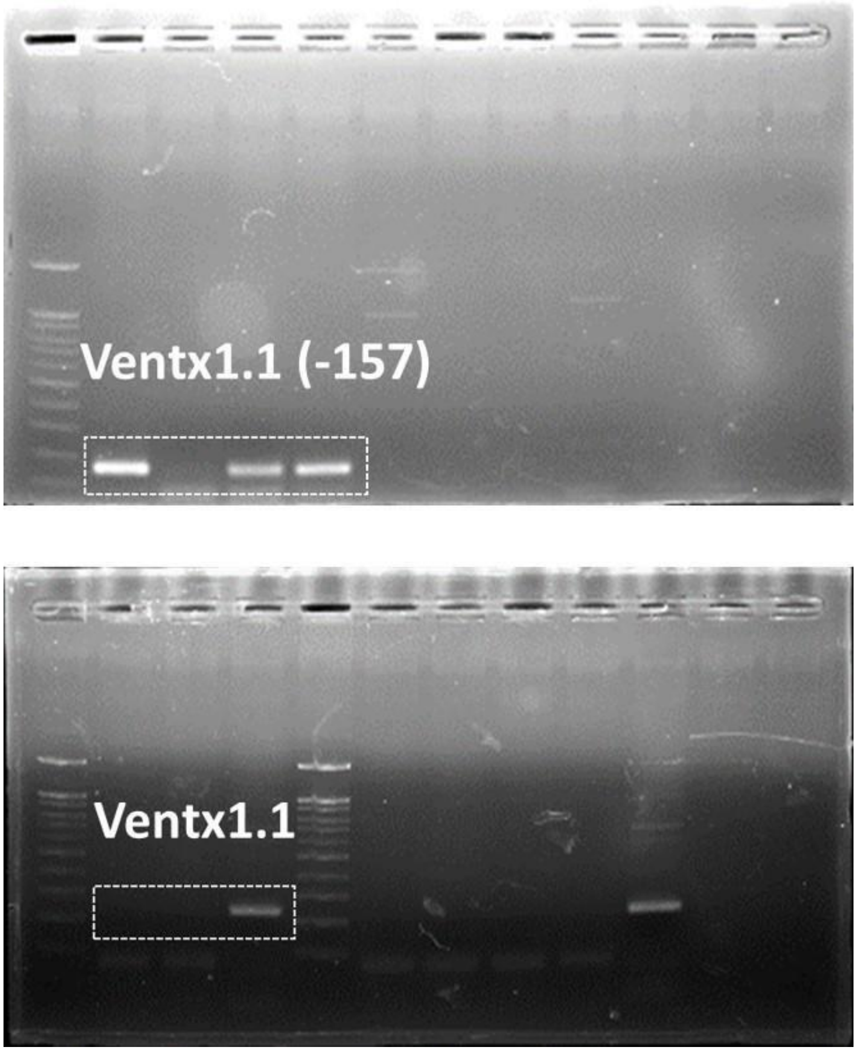

Figure 2f: Full gel images (Dashed boxes indicate the portion of gels included in the figures)

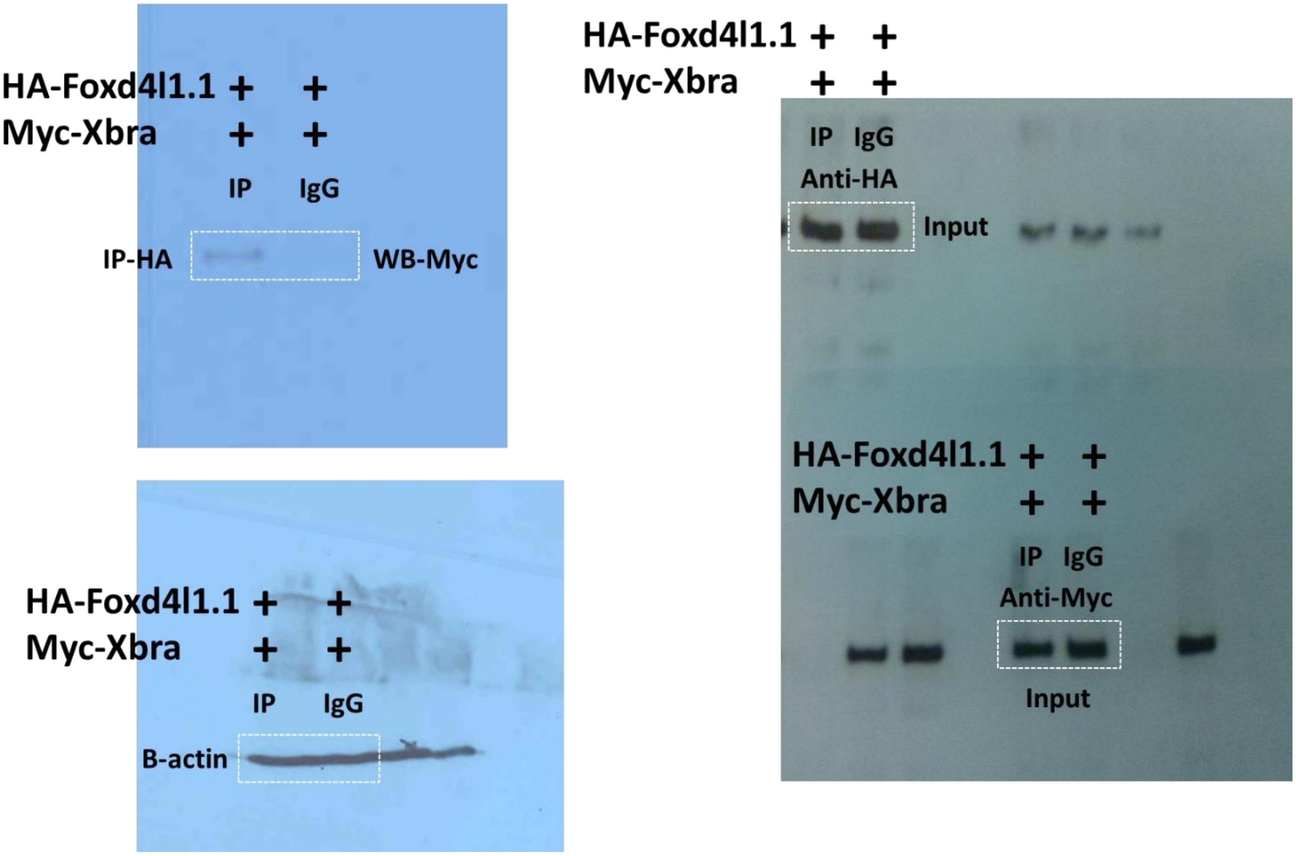

Figure 3b: Full film images (Dashed boxes indicate the portion of gels included in the figures)

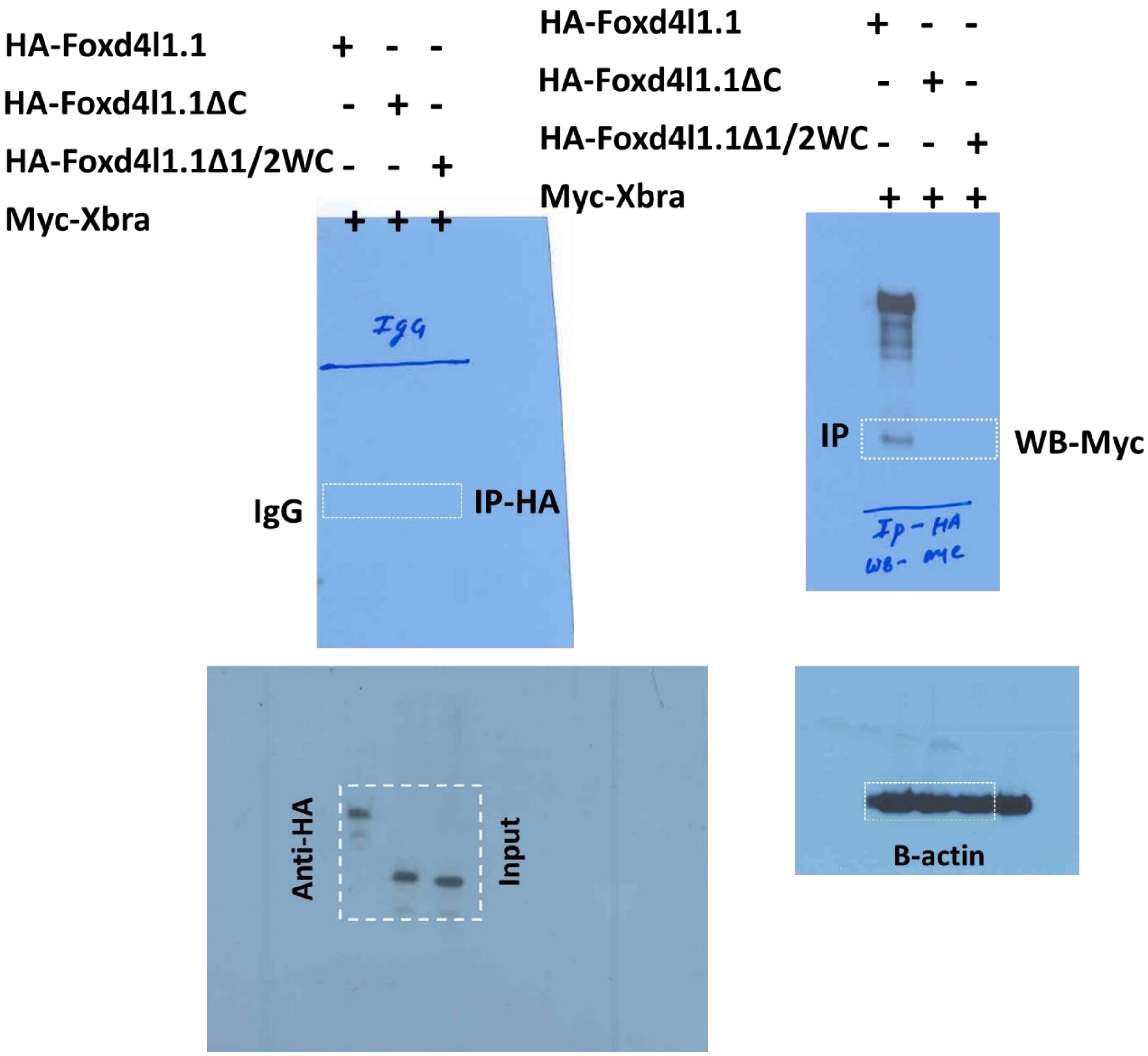

Figure 3d: Full film images (Dashed boxes indicate the portion of gels included in the figures)

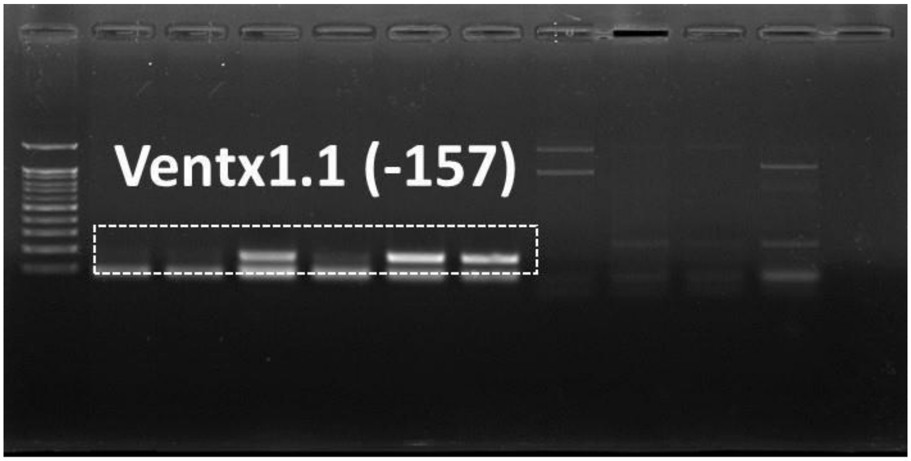

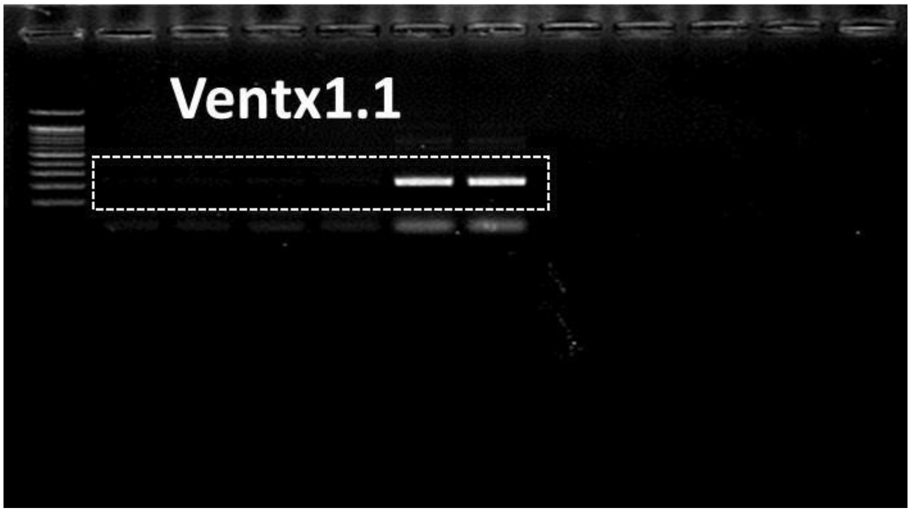

Figure 3e: Full gel images (Dashed boxes indicate the portion of gels included in the figures)

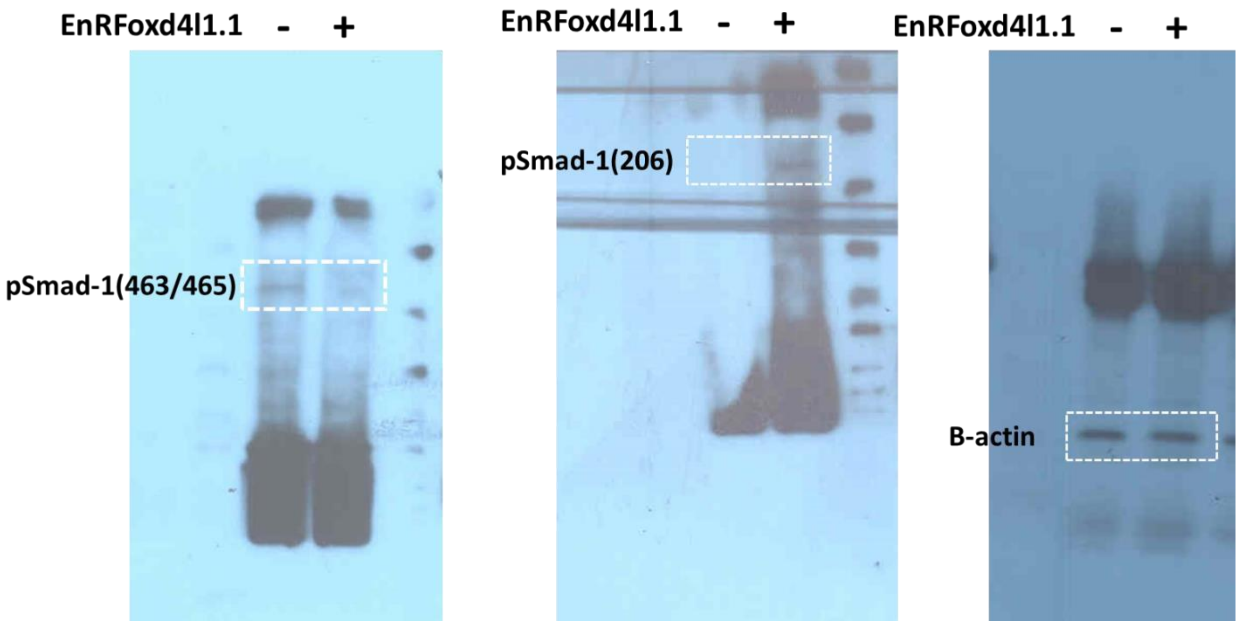

Figure 4b: Full film images (Dashed boxes indicate the portion of gels included in the figures)

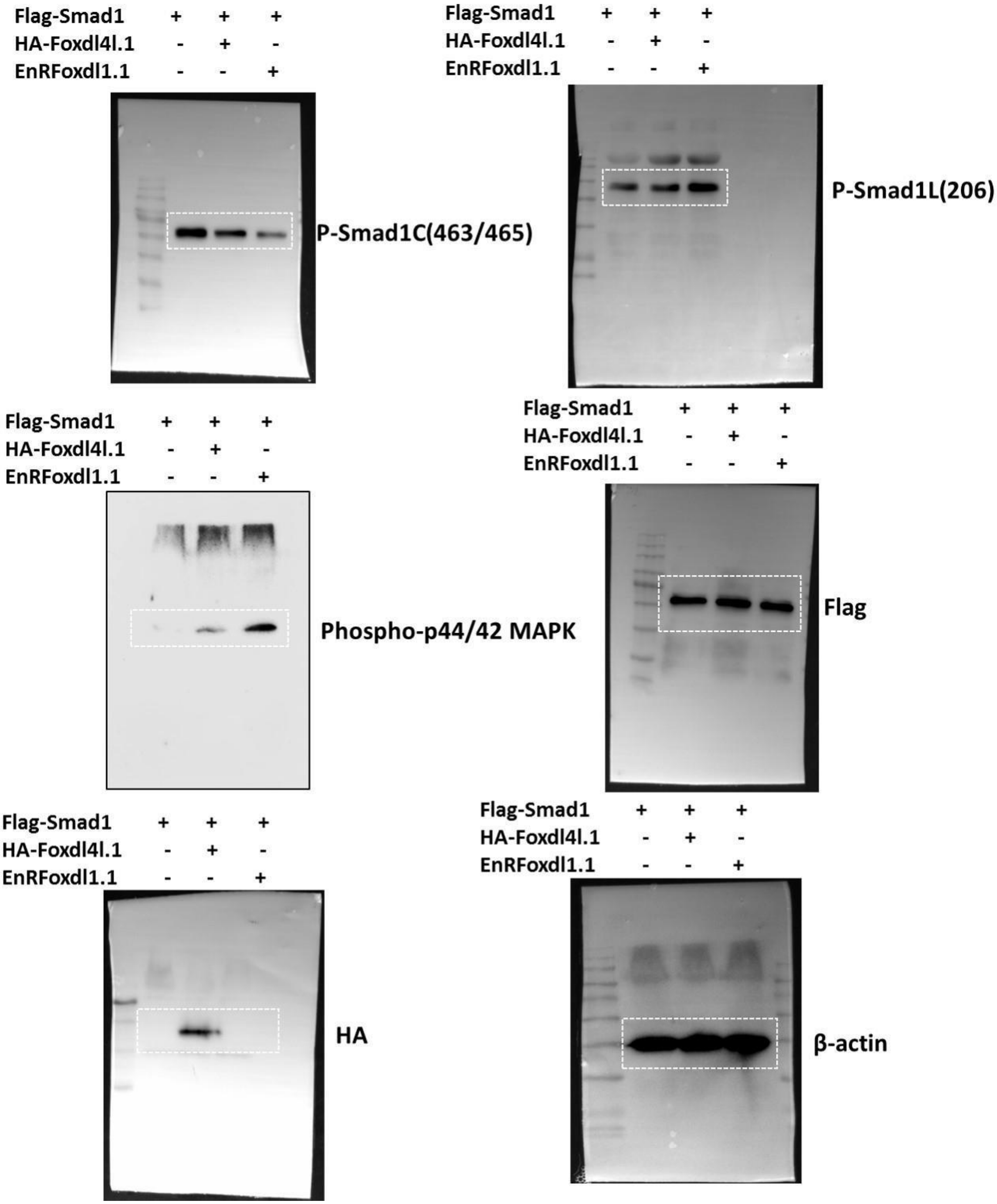

Figure 4c: Full membrane images (Dashed boxes indicate the portion of gels included in the figures)

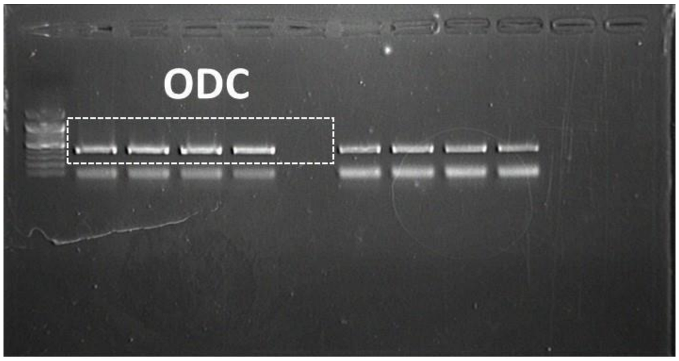

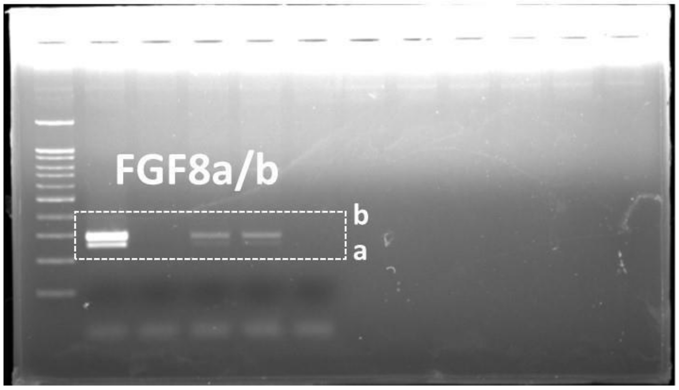

Figure 4e: Full gel images (Dashed boxes indicate the portion of gels included in the figures)

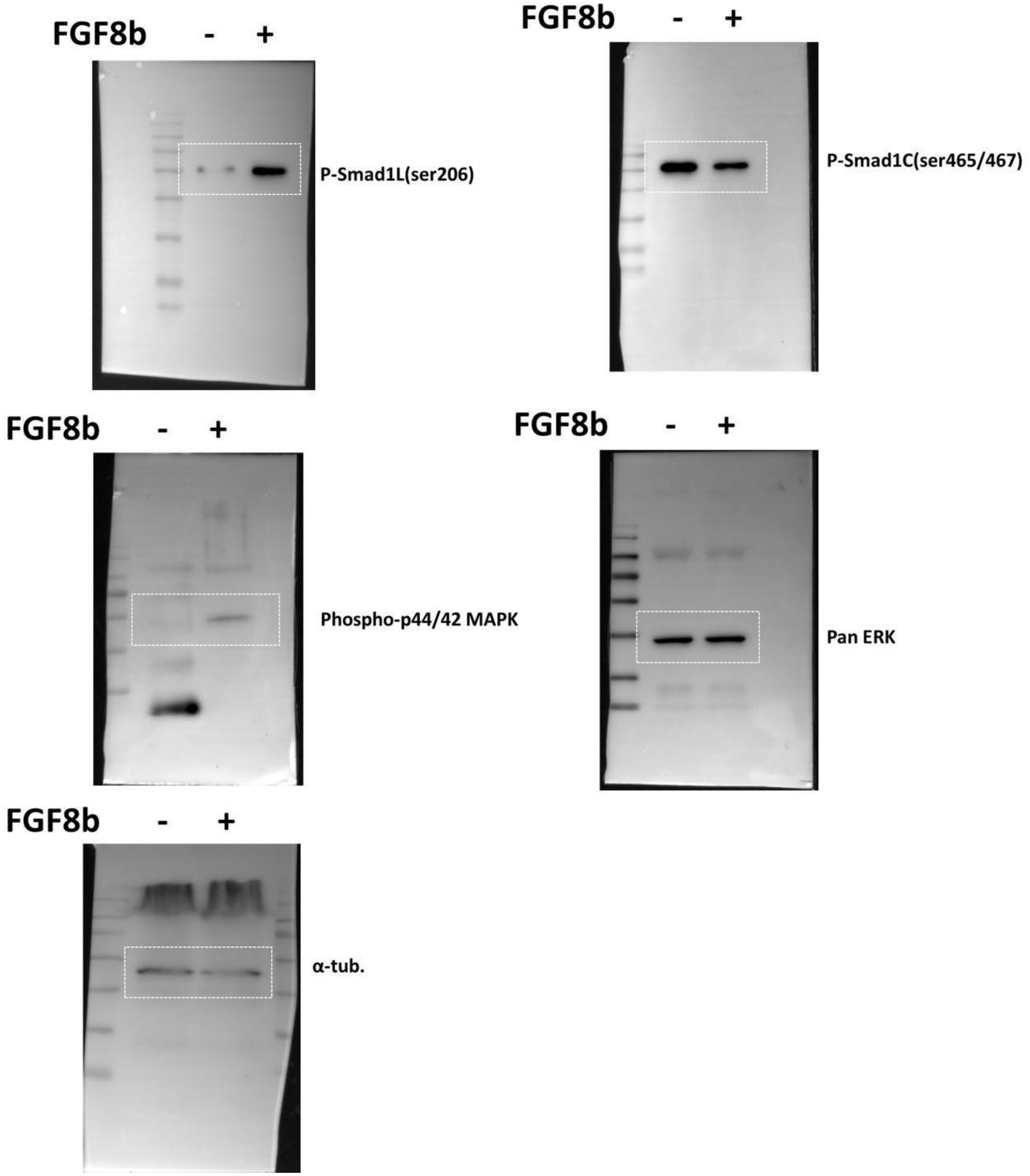

Figure 4f: Full membrane images (Dashed boxes indicate the portion of gels included in the figures)

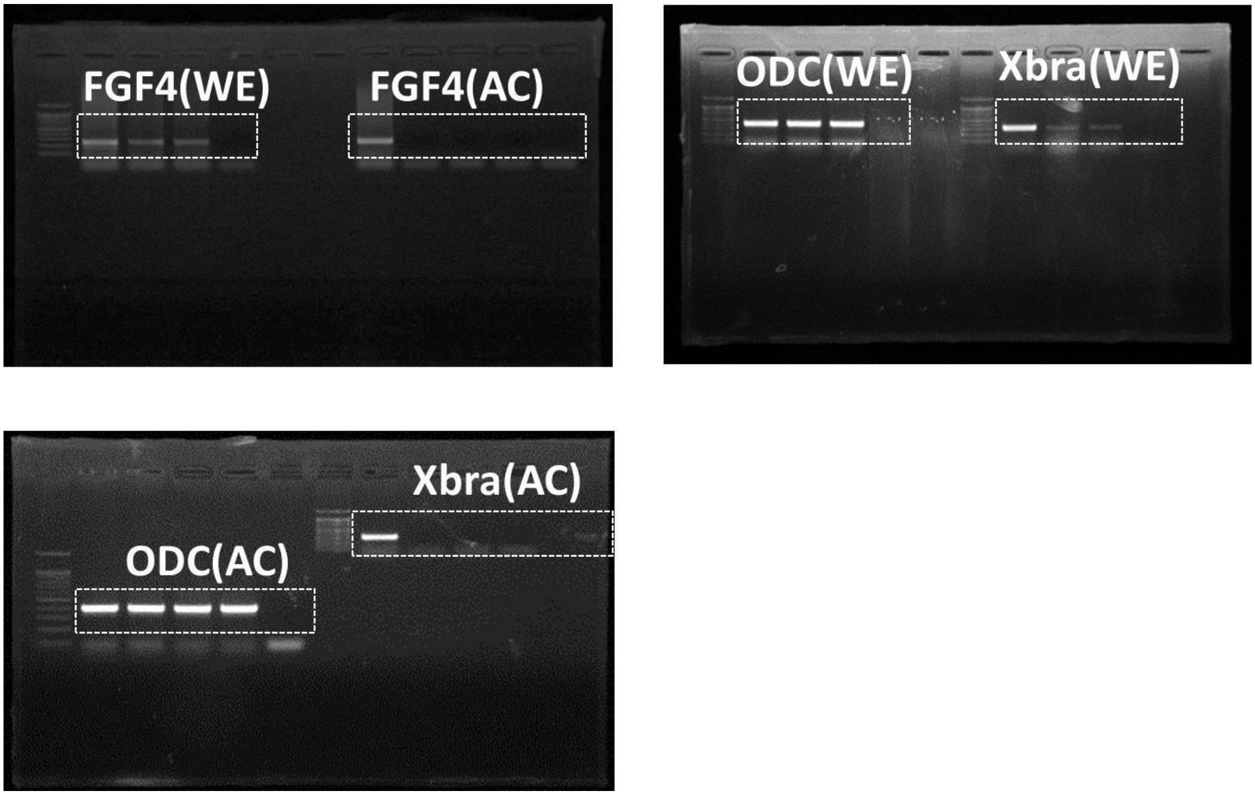

Figure 4h and i: Full gel images (Dashed boxes indicate the portion of gels included in the figures)

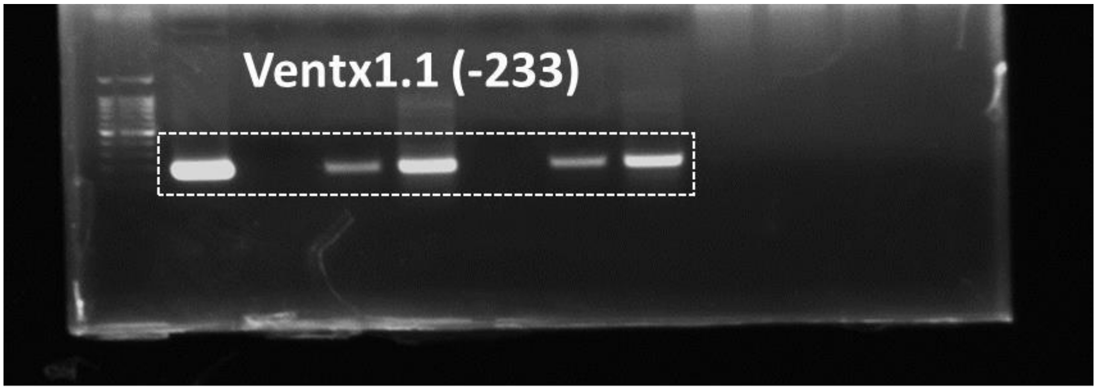

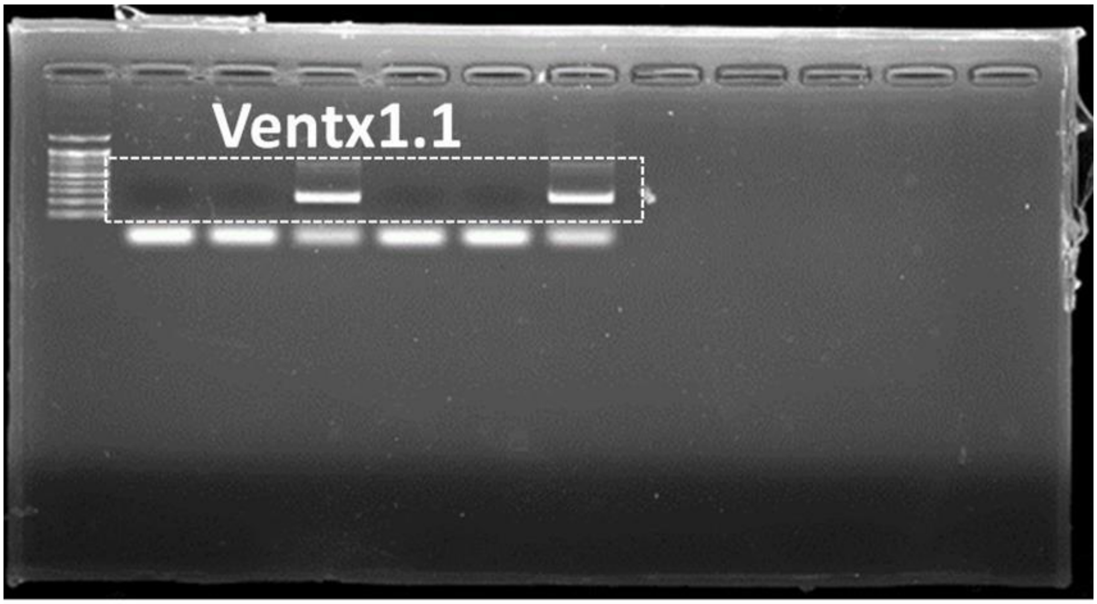

Figure 4j: Full gel images (Dashed boxes indicate the portion of gels included in the figures)

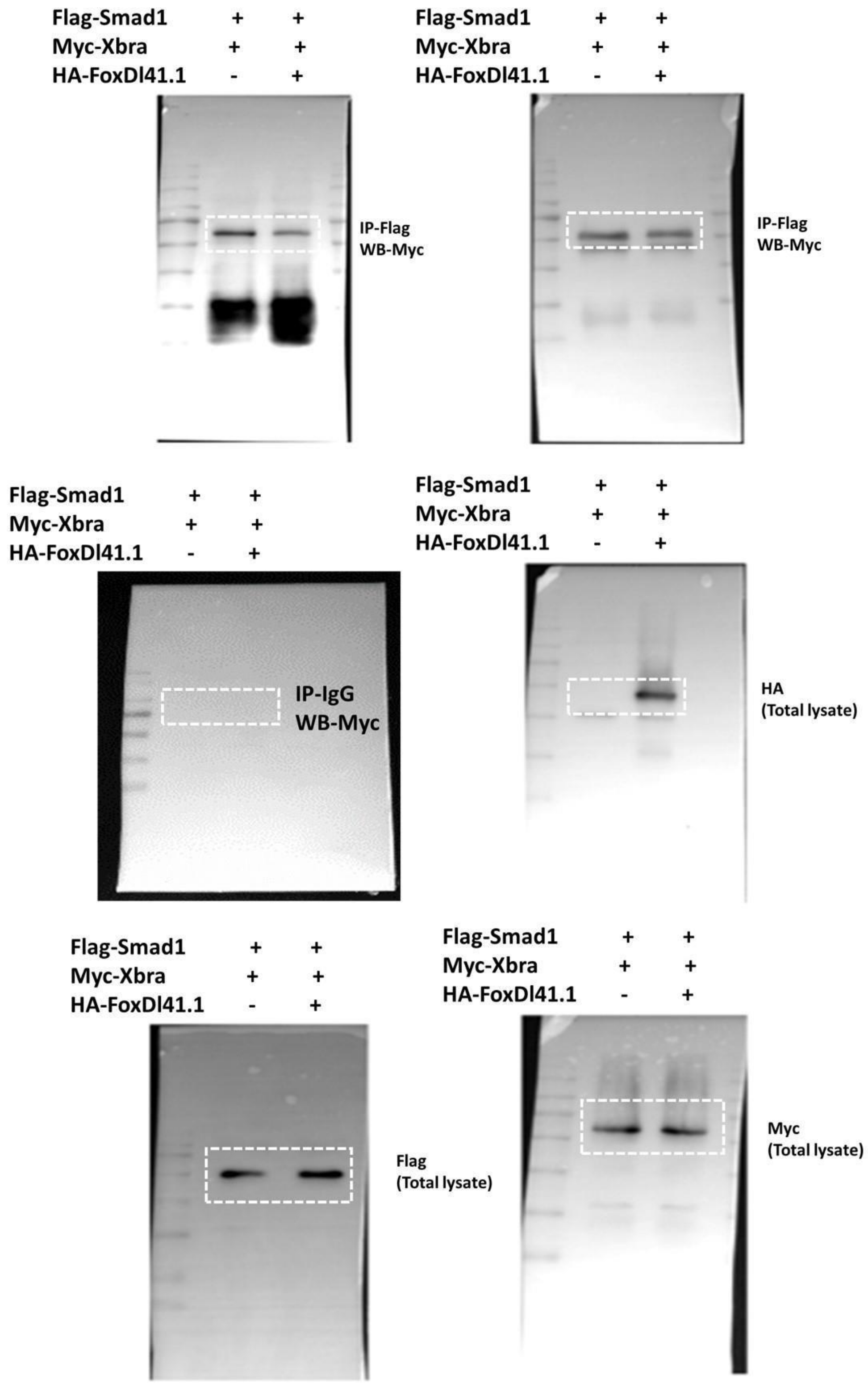

Figure 5b: Full membrane images (Dashed boxes indicate the portion of gels included in the figures)

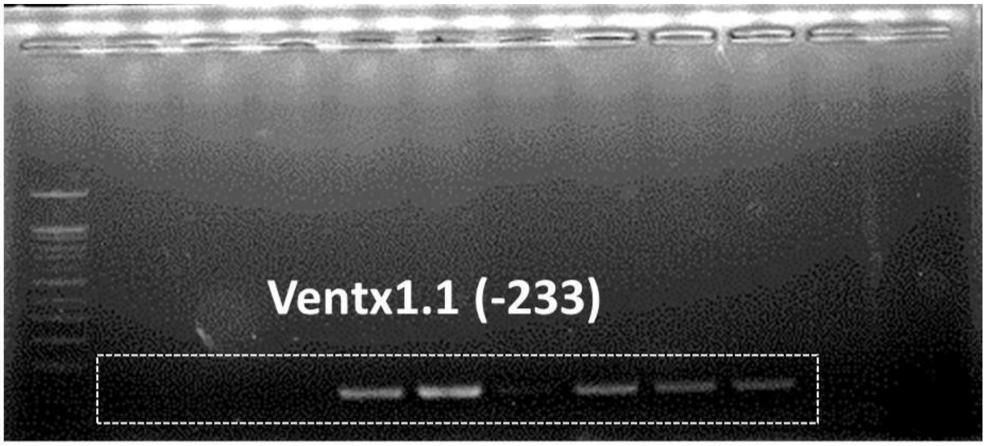

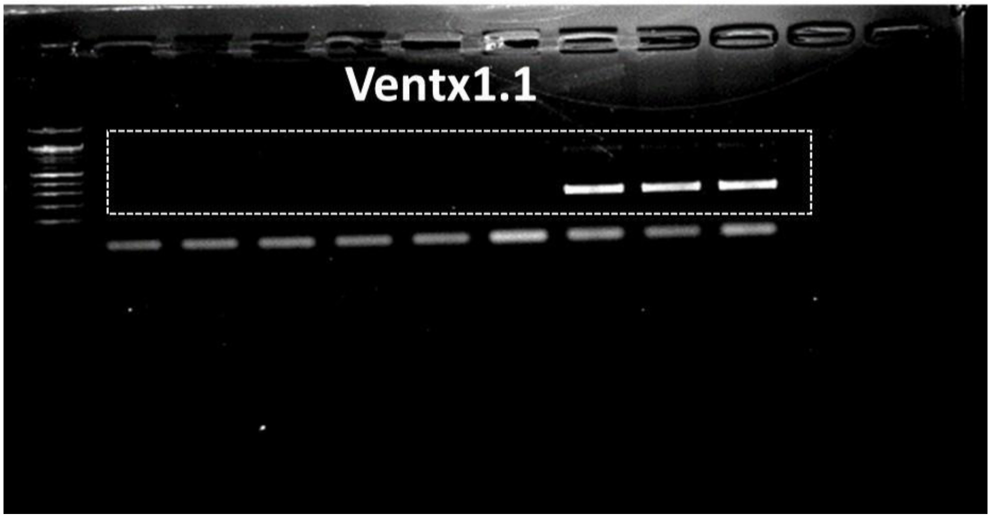

Figure 5c: Full gel images (Dashed boxes indicate the portion of gels included in the figures)

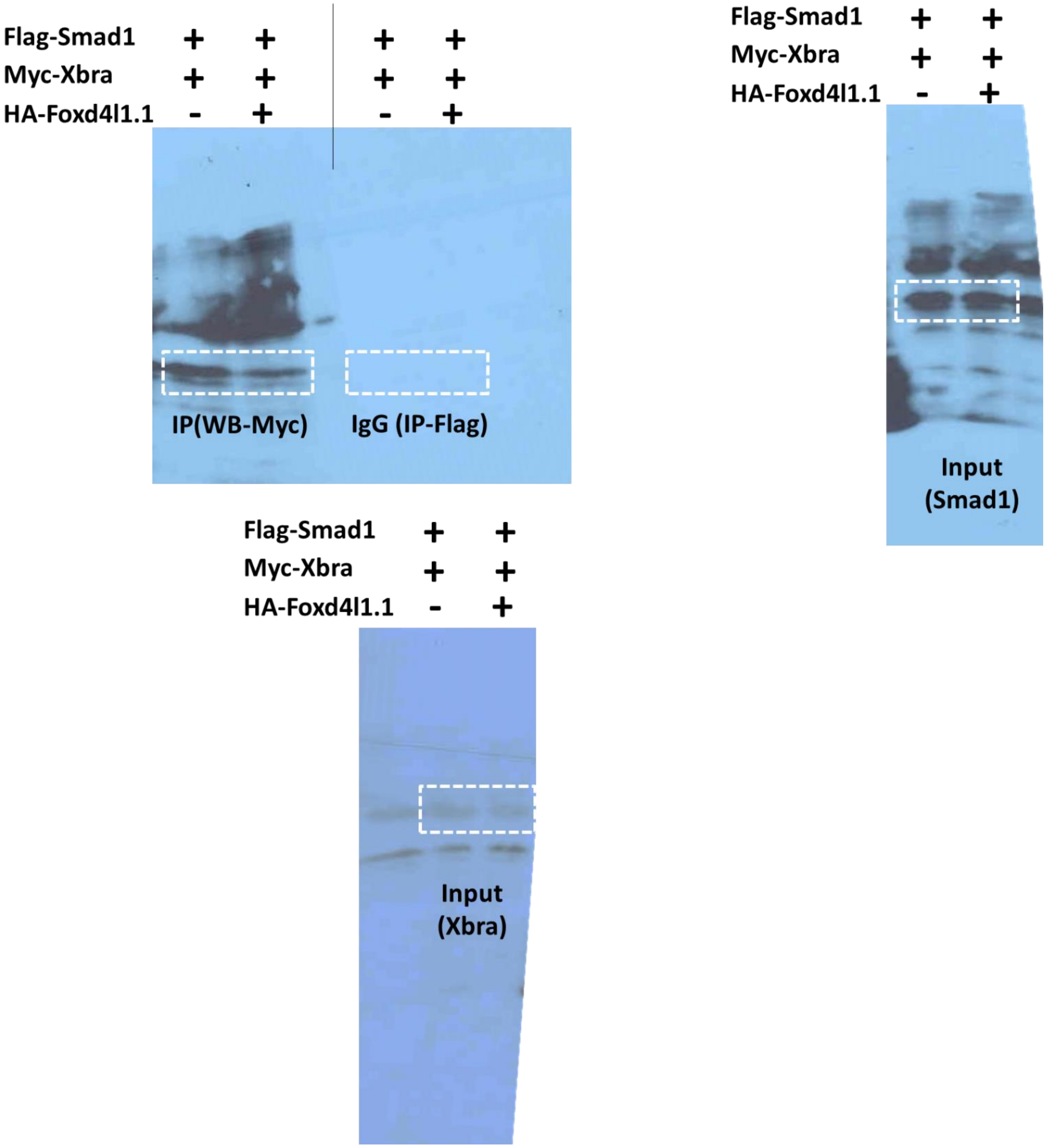

Supplementary Figure 4a: Full film images (Dashed boxes indicate the portion of gels included in the figures)

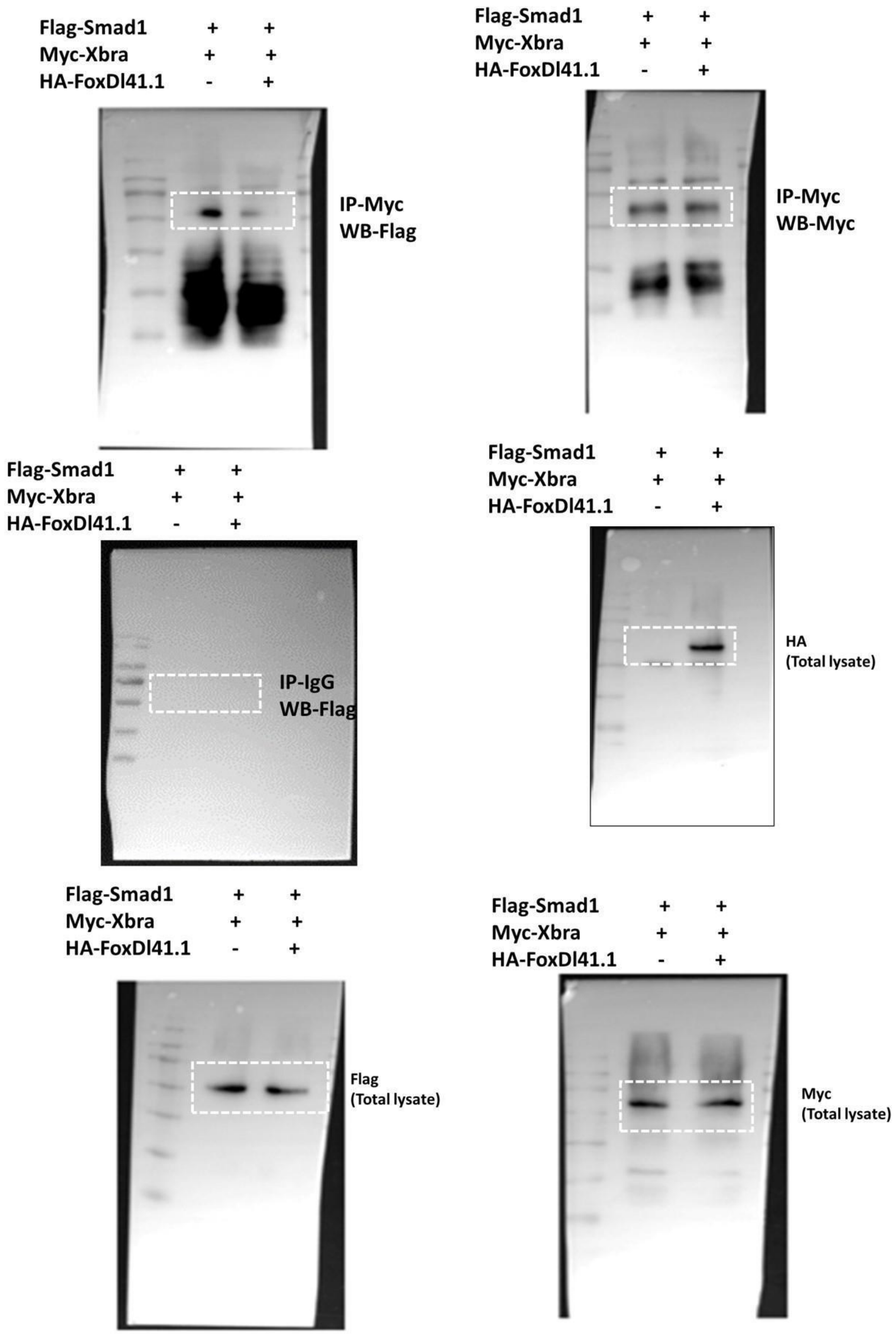

Supplementary Figure 4b: Full membrane images (Dashed boxes indicate the portion of gels included in the figures)
